# Human naïve B cells show evidence of anergy and clonal redemption following vaccination

**DOI:** 10.1038/s41541-025-01133-w

**Published:** 2025-05-14

**Authors:** Brian L. P. Dizon, Prasida Holla, Evan C. Mutic, Paul Schaughency, Susan K. Pierce

**Affiliations:** 1 Rheumatology Fellowship Training Program, National Institute of Arthritis and Musculoskeletal and Skin Diseases, National Institutes of Health, Bethesda, MD USA; 2 Laboratory of Immunogenetics, National Institute of Allergy and Infectious Diseases, National Institutes of Health, Rockville, MD USA; 3 Ryan White Center for Pediatric Infectious Diseases and Global Health, Indiana University School of Medicine, Indianapolis, Indiana USA; 4 Integrated Data Sciences Section, Research Technologies Branch, National Institute of Allergy and Infectious Diseases, National Institutes of Health, Bethesda, MD USA

**Keywords:** Peripheral tolerance, Antibodies, Immunological memory, Clonal selection, Humoral immunity, Immune tolerance, Somatic hypermutation

## Abstract

In an era of predicted emerging pandemics, the production of effective vaccines may require an in-depth understanding of the biology of human naive B (B_N_) cells. Here we provide evidence that the majority of B_N_ cells expressed CD73, an ecto-5’-nucleotidase often associated with immune cell suppression, and demonstrated features of anergy, including an IgM^low^IgD^+^ surface phenotype, reduced calcium flux in response to IgM crosslinking, and increased PTEN expression. Analysis of antibody sequences encoded by the inherently autoreactive V_H_4-34 heavy chain produced by plasmablasts seven days following influenza vaccination showed that in younger but not in older individuals, anergic B_N_ cells provided a reservoir of B cells capable of responding to vaccination by somatic mutation, resulting in diversification and loss of autoreactivity. These results suggest that effective human vaccines may require the ability to awaken or ‘redeem’ anergic B_N_ cells that can be repurposed to participate in pathogen-specific responses.

## Introduction

It is predicted that the world’s human populations have entered an era of emerging and recurring pandemics, a future in which our ability to produce highly effective, pathogen-specific vaccines that provide protection to individuals who have not acquired immunity to a pathogen or its variants will be critical. Reaching this goal will benefit from a more complete understanding of the characteristics of human naive B (B_N_) cells and the requirements for a vaccine to activate them.

At present, our knowledge of the composition of the human B_N_ compartment is limited, in part because most of what we know about the mammalian immune system has come from studies in inbred mice as recently reviewed^1,2^. For decades, mice provided models in which the basic mechanisms underlying the complex workings of the immune system were deciphered and the basic framework of the cellular and molecular mechanisms that underlie immune responses were established, providing critical insights necessary to translate this knowledge into therapies and vaccines. However, despite numerous important strides in human medicine based on insights gained in mouse models, predictions for human therapies and vaccines from mouse studies have more often than not been disappointing. Given large gaps in our knowledge of the human immune system, we undertook a direct investigation into human B_N_ cell biology with a view toward contributing to the future development of effective vaccines.

It is now clear that vaccine development must take into account that the human B_N_ cell repertoire contains not only B cells that express antibodies that are potentially protective against foreign pathogens or their variants, but also contains B cells that express self-reactive antibodies^3^. The process of V(D)J recombination by which a diverse B_N_ cell antibody repertoire is generated is inherently error-prone, inevitably resulting in the production of autoreactive B cells that are subsequently eliminated or inactivated. Elimination or inactivation of autoreactive B cells occurs at two key checkpoints. The first checkpoint, central tolerance^4^, functions in the bone marrow to eliminate B cells expressing high-affinity, self-reactive B cell receptors (BCRs) primarily by receptor editing and clonal deletion mechanisms. It is estimated that 75% of newly emerging immature bone marrow B cells in humans are self-reactive as determined by the generation of cloned antibodies and analysis of their binding to nuclear and cytoplasmic antigens in Hep-2 cell extracts and that this number was reduced by approximately half through central tolerance mechanisms^4,5^. Of note, we primarily cite the relevant literature from studies of human B cells and not similar studies in mice as these are often difficult to directly compare and mouse studies have been described in several excellent recent reviews^6^. The second checkpoint, peripheral tolerance, silences newly mature B_N_ cells that escaped central tolerance but express a relatively weak autoreactive BCR by triggering a state of hyporesponsiveness in these cells termed anergy^7,8^. Evidence for human B cell anergy was provided by a report of hyporesponsiveness to antigen in a significant fraction of human B cells expressing the inherently autoreactive heavy chain V_H_4-34, identified by the 9G4 anti-idiotype^9^. Because the precise surface marker phenotype of human autoreactive anergic B cells is currently unknown, estimates of the size of the human anergic B cell component have been variable. Screening of recombinant antibodies produced by human immature and B_N_ cells for reactivity to a defined set of self-antigens resulted in an estimate that ~20% of B_N_ cells are autoreactive and potentially anergic^4^. However, this result is likely an underestimate given the limited number of autoantigens tested. There is also compelling evidence that autoreactive anergic mature B_N_ cells express a shared phenotype, namely downregulation of IgM but not IgD BCRs resulting in an IgM^low^IgD^+^ phenotype^7,10,11^. For example, B_N_ cells with lower surface IgM levels (IgM^low^) were shown to be hyporesponsive to B cell receptor (BCR) crosslinking and have an increased frequency of autoreactive B cells as compared to B_N_ cells with higher surface IgM levels (IgM^+^)^7^. However, estimates of the size of the human anergic B cell pool based on IgM^low^ expression vary widely from 2.5% to 30% likely due to differences related to flow cytometry gating strategies and staining protocols^6,7,12^. More recently, elevated PTEN levels were found to be inversely correlated with surface IgM expression and calcium fluxes in response to BCR crosslinking. By using elevated PTEN as an alternative marker of B cell anergy, it was estimated that >40% of B_N_ cells may in fact be anergic^12^. Taken together, a large portion of the human B_N_ cell pool is comprised of anergic B cells, and further defining their surface marker phenotype will advance their study.

It has been proposed that anergic B cells are provided survival signals through both BAFF signaling and tonic signaling through the IgD BCR, allowing anergic B cells to serve as a reservoir for autoreactive B cells, the elimination of which could result in ‘holes’ in the B cell repertoire^3^. Thus, anergy, rather than permanently disposing of self-reactive B cells, appears to allow their survival and even their ‘clonal redemption’ and recruitment into pathogen-specific antibody responses as proposed by Goodnow and colleagues based on studies primarily in mice but also in humans^8,13^. At present, the fate and function of human anergic B cells remains unclear. It has been suggested that the very fact that the human immune system has evolved to maintain autoreactive B cells in an unresponsive state rather than eliminating them is evidence that anergic B cells are fated to provide a critical function in antibody responses^8^.

In this study, we took advantage of an established marker of human anergic B cells, namely IgM^low^IgD^+^ isotype expression, and the antibody that recognizes the inherently autoreactive Ig gene V_H_4-34, 9G4 anti-idiotype, to 1) identify and characterize naturally-occurring human anergic B cells by flow cytometry and 2) examine the outcome of V_H_4-34 sequences derived from anergic B cells in response to vaccination. Recently, a classification scheme was described^14^ based on cell surface marker expression that allows segregation of all human peripheral blood B cells into 10 subsets. We used this classification scheme to characterize in detail human B_N_ cells in the peripheral blood of healthy younger and older individuals. Our results provide a comprehensive view of the human B_N_ cell compartment. Rather than being composed of resting, antigen-inexperienced B cells, the majority of B_N_ cells express an IgM^low^ IgD^+^ phenotype associated with human B cell anergy and can be distinguished by the expression of CD73, an ecto-5’-nucleotidase that catalyzes ATP to adenosine, often associated with immune cell suppression^15^. Analyses of peripheral blood B cell antibody sequences produced by plasmablasts in individuals seven days following vaccination provided evidence that anergic B_N_ cells may serve as a reservoir of B_N_ cells capable of responding to vaccination by somatic mutation (SM) and diversification. These results suggest that effective vaccines in humans may require the ability to awaken or ‘redeem’ anergic B_N_ cells that can be repurposed to participate in a pathogen-specific response.

## Results

### Identification of human peripheral blood B cell subsets, including B_N_ cells, by flow cytometry

**Fig. 1:**
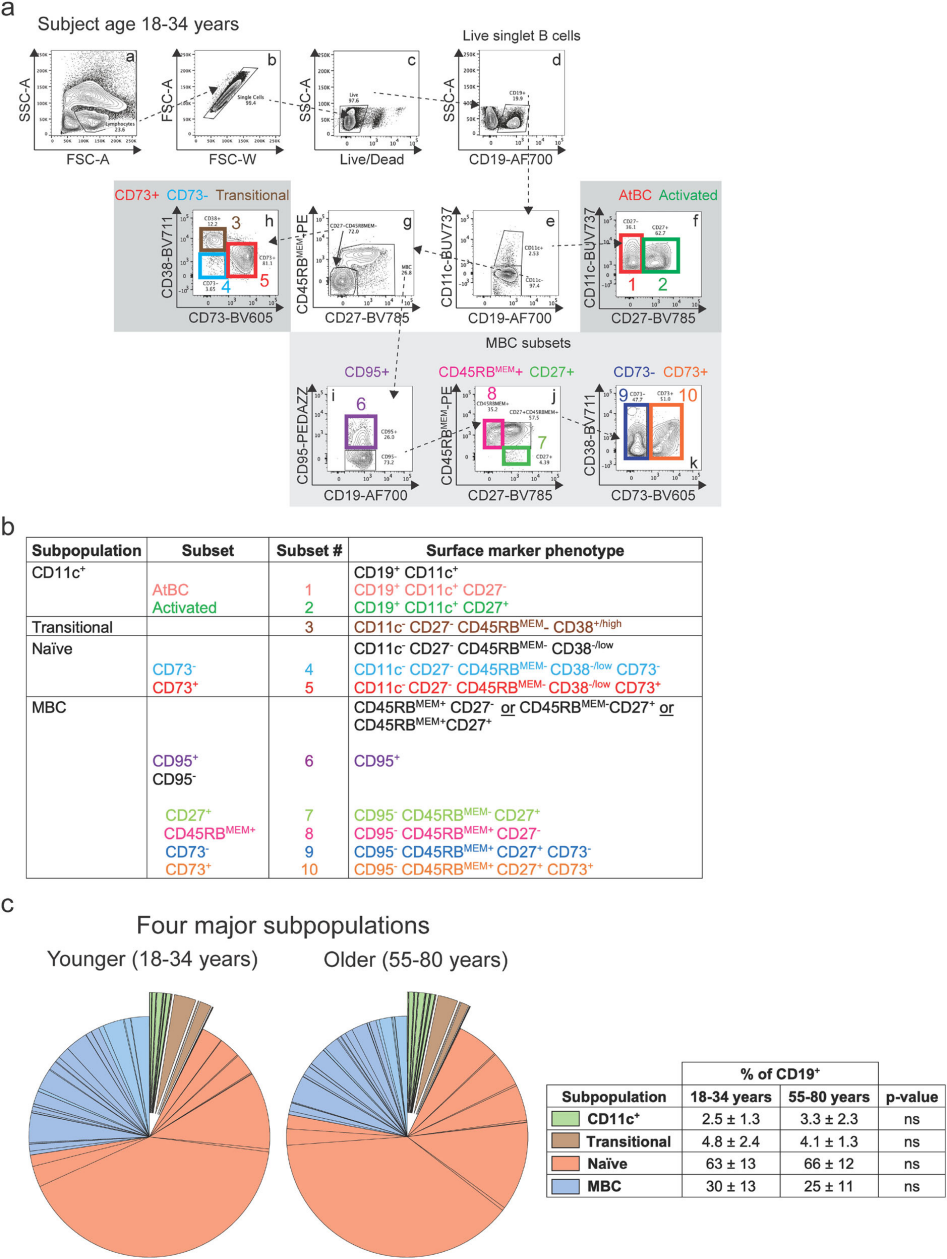
Flow cytometric identification of the known human B cell subsets. **a** The 10 B cell subsets in peripheral blood are color-coded and numbered in the flow cytometry gating strategy for a representative 18–34 year old human subject in the study. **b** Summary table of the cell surface marker phenotypes for the 10 B cell subsets in human peripheral blood. **c** Pie charts depicting the four major subpopulations in human peripheral blood of 18–34 year (younger) and 55–80 year (older) individuals. The table summarizes the percentages of each subpopulation (mean ± SD) of 15 individuals in the 18–34 years and 12 in the 55–80 year age groups. Statistical significance was determined by Mann-Whitney test. ns= not significant.

To characterize human peripheral blood B_N_ cells we used a recently described flow cytometry-based classification scheme that segregates human peripheral blood B cells into 10 unique subsets^14^. The advantage of a flow cytometry-based assay is that it allows quantification of the cell surface expression of individual markers, providing a measure of the variability of the expression of key B cell markers within a subpopulation or subset. To better understand the characteristics of human B cells subpopulations and subsets over a lifetime, we analyzed and compared peripheral blood B cells from both younger and older individuals obtained from the NIH Blood Bank^16^. The demographic information of the subjects analyzed are summarized in Supplementary Tables 1 and 2. Representative flow cytometry plots are given for the peripheral blood B cells of a younger individual (18–34 years) (Fig. 1a). As shown, live singlet CD19^+^ B cells were segregated into two subpopulations: CD11c^+^ and CD11c^−^. CD11c^+^ cells were further segregated into two relatively equal sized subsets based on CD27 expression: CD11c^+^CD27^−^ termed atypical B cells (AtBCs)^17^ (Fig. 1b, subset 1) and CD11c^+^CD27^+^ (activated B cells) (Fig. 1b, subset 2). CD11c^−^ cells were further segregated into a CD27^−^CD45RB^MEM−^ subpopulation that was identified as transitional B cells by the expression of CD38 (CD11c^−^CD27^−^CD45RB^MEM −^ CD38^+^) (Fig. 1b, subset 3) or as B_N_ cells by low to absent CD38 expression (CD11c ^−^ CD27 ^−^ CD45RB^MEM −^ CD38^low/ −^ ) (Fig. 1b). B_N_ cells were further separated into two subsets distinguished by CD73 expression, CD73^-^ (Fig. 1b, subset 4) and CD73^+^ (Fig. 1b, subset 5). CD73 is an ecto-5’-nucleotidase that catalyzes the last step in the extracellular metabolism of ATP to form adenosine and is a key component of purinergic signaling, a fundamental mechanism used by all cells to control internal activities and interact with the environment^18^. Thus, we identified two human peripheral blood B_N_ cells subsets defined as CD11c ^−^ CD27 ^−^ CD45RB^MEM −^ CD38 ^− /low^ and either CD73^+^ or CD73 ^−^ ^14^.

Continuing the analysis, memory B cells (MBCs) were defined as either CD45RB^MEM+^CD27^-^, CD45RB^MEM+^CD27^+^, or CD45RB^MEM −^ CD27^+^, and MBCs were further separated based on CD95 expression into one CD95^+^ subset (Fig. 1b, subset 6) and four CD95 ^−^ subsets: CD95 ^−^ CD45RB^MEM-^CD27^+^ (Fig. 1b, subset 7), CD95 ^−^ CD45RB^MEM+^CD27 ^−^ (Fig. 1b, subset 8), and CD95 ^−^ CD45RB^MEM+^ CD27^+^, that was further separated into two subsets based on CD73 expression, CD73 ^−^ (Fig. 1b, subset 9) and CD73^+^ (Fig. 1b, subset 10). The same analysis was carried out on peripheral blood cells from older individuals (55-80 years) (Supplementary Fig. 1a). A quantification of the percent of total peripheral blood CD19^+^ B cells that were contained in each of the four major human B cell subpopulations, namely B_N_ cells, MBCs, CD11c^+^ B cells, and transitional B cells for younger (18–34 years) and older (55–80 years) individuals showed these to be highly similar between age groups (Fig. 1c). As shown, the majority of CD19^+^ peripheral blood B cells from both younger and older individuals were B_N_ cells ( ~ 63-66%), MBCs accounted for the next largest subpopulation ( ~ 25–30%) and transitional B cells and CD11c^+^ B cells represented minor populations of 4–5% and 2–3%, respectively.

### Characterization of peripheral blood B_N_ cells by flow cytometry

**Fig. 2:**
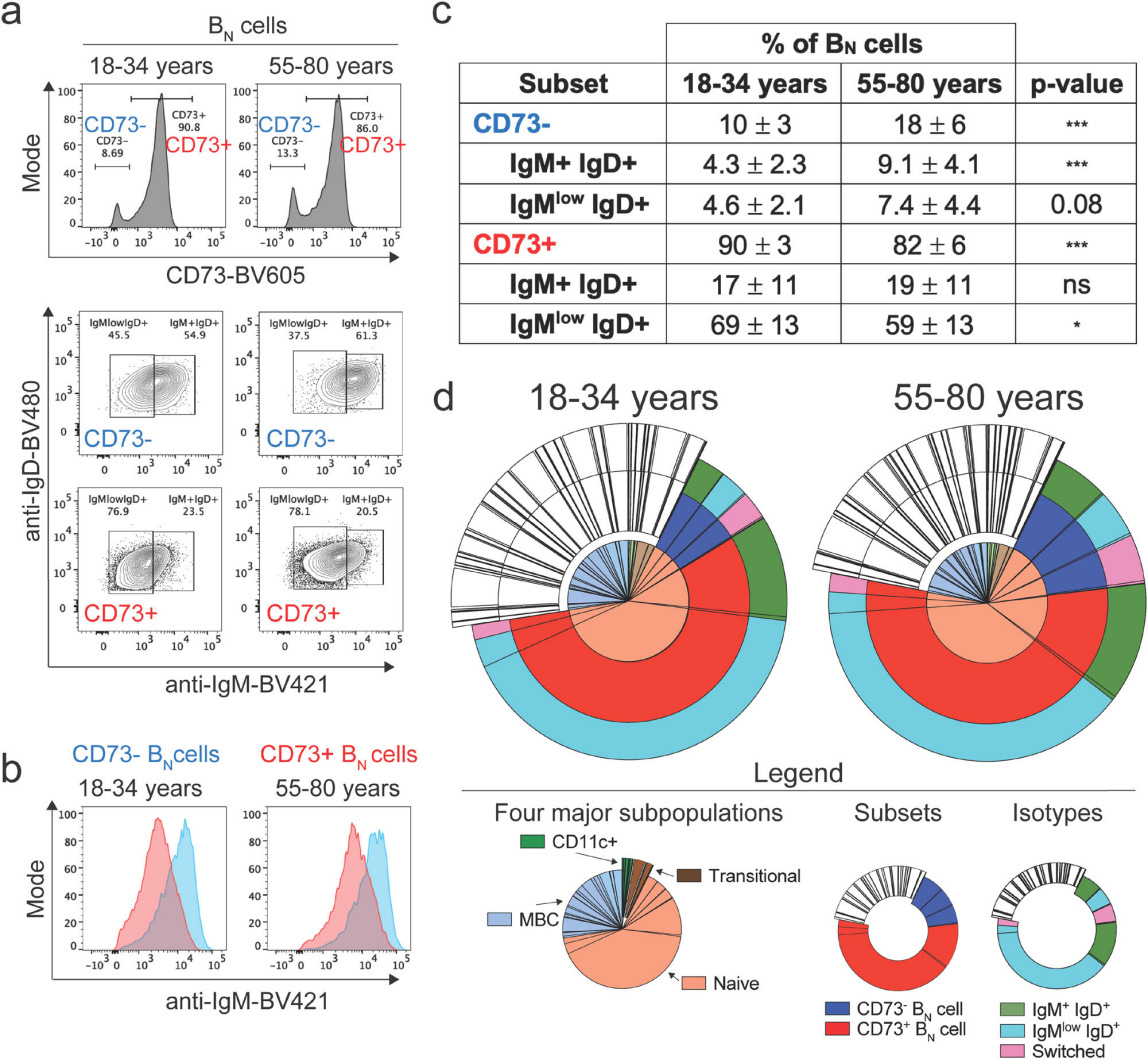
Percentages of B_N_ cell subsets in peripheral blood according to cell surface markers and BCR isotype. **a** Representative flow cytometry plots depicting CD73 expression in B_N_ cells and identification of IgM^+^IgD^+^ and IgM^low^IgD^+^ B_N_ cells in two individuals from each of the 18–34 and 55–80 year age groups. **b** Representative histograms depicting surface IgM staining in CD73^-^ and CD73^+^ B_N_ cells from 18–34 and 55-80 year old subjects. **c** Table summarizing the percentages of IgM^+^IgD^+^ and IgM^low^IgD^+^ B cells within the CD73^-^ and CD73^+^ B_N_ cell subsets (mean ± SD). The data represent 15 individuals in the 18–34 years and 12 in the 55–80 year age groups. Statistical significance was determined by Mann-Whitney test. *p < 0.05, ***p < 0.001, ns= not significant. **d** Target plots summarizing the composition of B_N_ cells based on CD73 expression and isotype expression in 18–34 and 55–80 year old individuals. For simplicity, only the CD73^-^ and CD73^+^ B_N_ cell subsets are color-coded. The complete CD11c^+^, transitional, and MBC subpopulations are shown in white and given in Supplementary Fig. 1.

Given that CD73 expression distinguished two human B_N_ subsets, we quantified the percent of B_N_ cells that were CD73^+^ versus CD73 ^−^ . The CD73 expression levels of B_N_ cells were bimodal with the vast majority of cells (~80–90%) expressing CD73 (CD73^+^) and the remainder (10–18%) showing little to no CD73 expression (CD73 ^−^ ) (Fig. 2a).

We also determined the isotype expression within these two CD73 subsets as a nearly universal feature of human anergic B cells is their IgM^low^IgD^+^ phenotype^7^. To do so, IgD-specific BV480-labeled antibodies (anti-IgD-BV480) and IgM-specific BV421-labeled antibodies (anti-IgM-BV421) were added to the antibody panel used to characterize the human B cell subsets. A flow cytometry gating strategy performed on each individual in the study is shown in Supplementary Fig. 1b as steps 1-3. In step 1, among total live CD19^+^ B cells in peripheral blood, four groups were distinguished according to expression levels of IgM and IgD. The first group of CD19^+^ cells appeared to be switched in being negative for IgM and IgD. Among the remaining CD19^+^ B cells that expressed IgM and/or IgD, cells that expressed higher levels of IgM (IgM^+^) were divided into two groups according to the presence of IgD and named IgM^+^IgD^-^ and IgM^+^IgD^+^. The fourth group of cells which were IgD^+^ but expressed low IgM levels were termed IgM^low^IgD^+^. In Step 2, after defining these four groups in peripheral blood in each individual, the gates were applied to B_N_ cells that were CD73^-^ or CD73^+^ (Supplementary Fig. 1b). Shown are representative flow cytometry plots of CD73, IgM, and IgD expression for a younger versus an older individual (Fig. 2a). In Step 3, among human B_N_ cells that express surface IgM along a continuum^19^, anti-IgD-

BV480 and anti-IgM-BV421 identified two subsets among both CD73 ^−^ and CD73^+^ B_N_ cells, namely IgM^low^IgD^+^ and IgM^+^IgD^+^ (Supplementary Fig. 1b, Fig. 2a). Overlay of IgM expression in B_N_ cell subsets showed that CD73^+^ B_N_ cells expressed lower surface IgM compared to CD73^-^

B_N_ cells (Fig. 2b). Shown are the average percents of total B_N_ cells that expressed CD73 and were either IgM^+^ or IgM^low^, quantified from analyses of peripheral blood B_N_ cells from 15 younger and 12 older individuals (Fig. 2c). The largest subpopulation was CD73^+^ IgM^low^IgD^+^ that accounted for well over half of CD19^+^ B_N_ cells in both younger (69%) and older (59%) individuals (Fig. 2c). These data are also displayed in target plots for both younger (18–34 years) and older (55–80 years) individuals (Fig. 2d). The center of the target plots shows the four major human subpopulations (B_N_ cells, MBCs, CD11c^+^, and transitional cells) as a percent of total CD19^+^ cells. Shown in the first concentric color-coded ring is the percent B_N_ cells that are CD73^+^ or CD73^-^, and shown in the second concentric ring is the percent of B_N_ cells that are IgM^+^IgD^+^, IgM^low^IgD^+^, and switched. The small number of switched cells that were not considered to be B_N_ cells were excluded from further B_N_ cell analyses. The expression of CD73 and isotypes were similar among younger and older individuals, with the exception of the percent of CD73 ^−^ cells, which was smaller in younger individuals as compared to the older (10% versus 18%) (Fig. 2c). Similar subset analyses were also carried out for the remaining three major subpopulations (MBCs, CD11c^+^, and transitional), and these data were displayed separately (Supplementary Fig. 1c, d).

### The relationships between CD73, BCR isotype expression, and anergy in B_N_ cells

**Fig. 3:**
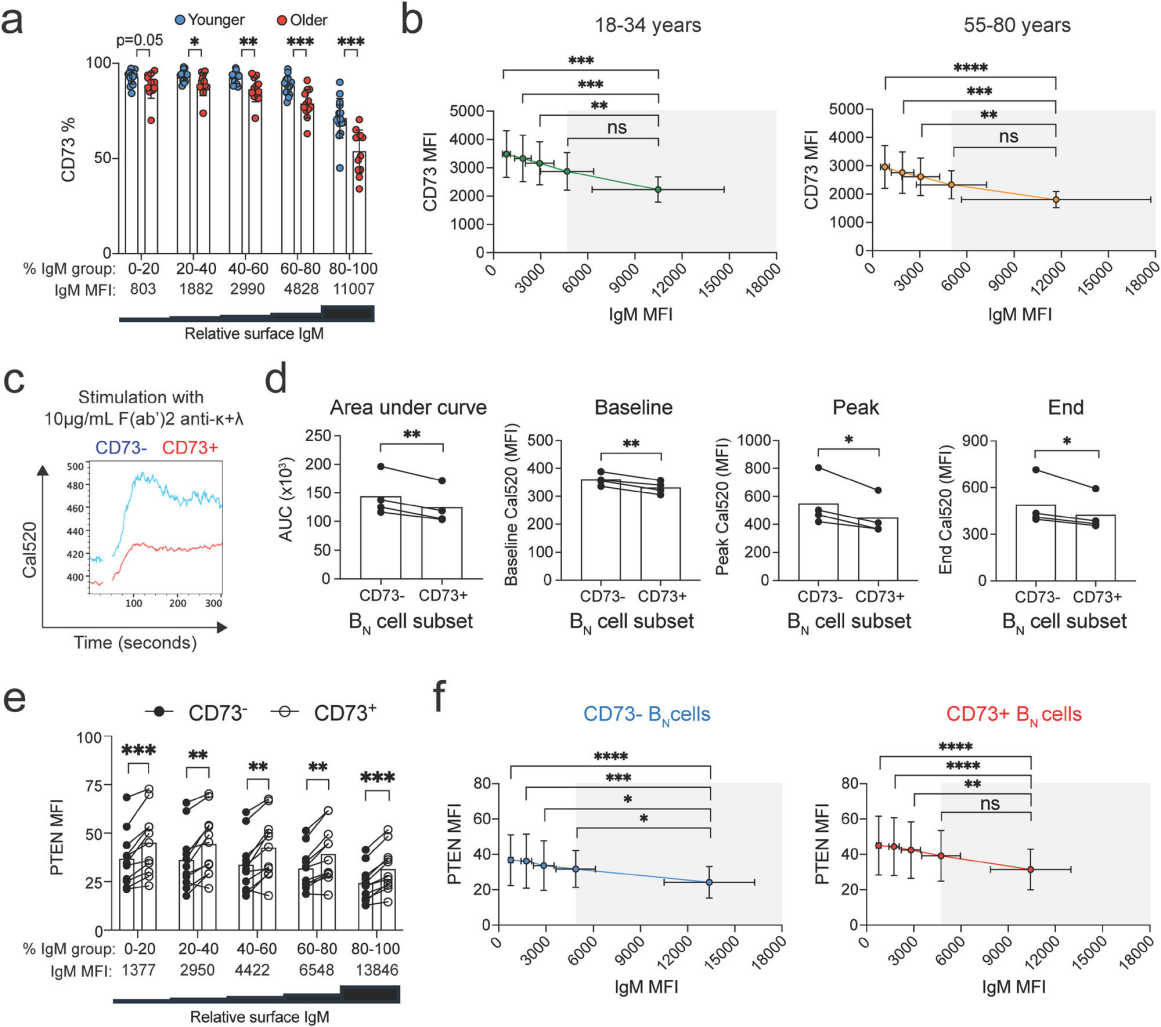
IgM expression, calcium fluxes, and intracellular PTEN levels by CD73^-^ and CD73^+^ B_N_ cells. **a** Relationship between relative surface IgM expression in 15 younger (18–34 years) versus 12 older (55–80 years) individuals as measured by percentage of CD73^+^ B_N_ cells expressing IgM within a given percent range (0–20, 20–40, 40–60, 60–80, and 80–100). Relative surface IgM expression is shown as MFI. Statistical significance was determined by Mann-Whitney test, *p < 0.05, **p < 0.01, and ***p < 0.001. **b** Relationship between CD73 expression and IgM expression among CD73^+^ B_N_ cells from 15 younger (18-34 years) versus 12 older (55–80 years) individuals. Statistical significance was determined by paired Wilcoxon test, **p < 0.01, ***p < 0.001, ***p < 0.0001, ns=not significant. Calcium fluxes of CD73^-^ (blue) and CD73^+^ (red) B_N_ cells to 10 μg/mL anti-κ+λ F(ab’)2 (**c**), and a summary of area under the curve (AUC), baseline, peak, and end in four healthy individuals (**d**). Statistical significance was determined by paired Wilcoxon test, **p < 0.01, ***p < 0.001. Comparison of PTEN expression between CD73^-^ and CD73^+^ B_N_ cells (**e**) and PTEN and surface IgM expression (**f**) from 10 individuals. Statistical significance was determined by paired Wilcoxon test, **p < 0.01, **p < 0.01, ***p < 0.001, ***p < 0.0001, ns=not significant.

We next compared the levels of surface expression of both IgM and CD73 for all CD73^+^ B_N_ cells from peripheral blood of the 15 younger and 12 older individuals. To do so, for each individual, B_N_ cells were binned based on IgM expression into five equal groups of equal size, each containing 20% of the B_N_ cells (Supplementary Fig. 2a). Then individuals were grouped according to age and their surface IgM levels compared. No differences in surface IgM expression of B_N_ cells in any of the five groups were observed between younger and older individuals (Supplementary Fig. 2b, c). For each of the five groups, the expression of CD73 was determined by quantifying the percentage of CD73^+^ B_N_ cells in each group (Fig. 3a) as well as the average MFI for each of the five IgM groups (Fig. 3b). These data are displayed showing the relationship between the levels of IgM and CD73 expression for each individual in each age group (Fig. 3a) as well as the average of all individuals in each group (Fig. 3b). Of note, the range of IgM expression was relatively large (MFIs of 803–11,007) (Fig. 3a, b), reflecting expression of surface IgM over a continuum^19^. In contrast, CD73 expression varied only over a relatively narrow range (MFIs 1500–3500) for both younger and older individuals (Fig. 3b). The analyses showed that the expression CD73 decreased with increased expression of IgM for younger and older individuals (Fig. 3a, b). The levels of IgM expression in the first four groups, which accounted for 80% of the CD73^+^ B_N_ cells, were more similar to each other (MFIs of 803–4828), but significantly less than that of B_N_ cells in the fifth group that expressed the highest levels of IgM (MFI 11,007) (Fig. 3a, b). Taken together, these data provide evidence that 80% of human CD73^+^ B_N_ cells express the anergic phenotype, IgM^low^IgD^+^, and 20% do not. Of interest, the levels of IgM expression in each group of IgM^+^ cells were similar in the younger and older (Supplementary Fig. 2b, c), but the percent of CD73 expression was significantly lower in the older B_N_ cells (Fig. 3a), suggesting that CD73 may function differently in the older versus the younger.

A key question to address is whether the IgM^low^IgD^+^ subset of peripheral blood human B_N_ cells identified was indeed functionally anergic. The cellular and molecular mechanisms underlying anergy in human B cells are only poorly understood, particularly as compared to mouse B cells^6,7,10^. To gain insight to the anergic state of human B_N_ cells, we evaluated the ability of the two different B_N_ subsets, as defined by CD73 expression, to flux calcium in response to BCR crosslinking. To do so, IgM-expressing B_N_ cells were purified from healthy individuals (Supplementary Table 3), labeled with Fab anti-IgM and anti-IgD that does not lead to BCR crosslinking, as well as fluorescently labeled anti-CD73 antibody to distinguish CD73 ^−^ and CD73^+^ B_N_ cells (Supplementary Fig. 3a, b). Labeling was carried out under conditions in which anti-CD73 antibody did not activate B_N_ cells (Supplementary Fig. 4a). The labeled B_N_ cells were then treated with 10μg/mL F(ab’)_2_ anti-κ + λ F(ab’)_2_ or F(ab’)_2_ anti-IgM to crosslink the BCRs to trigger calcium flux responses (Supplementary Fig. 4b). BCR labeling was stable over the time period the B_N_ cells were prepared and analyzed (Supplementary Fig. 4c-e). We observed a highly diminished calcium flux in CD73^+^ B_N_ cells versus CD73 ^−^ B_N_ cells when calcium levels were quantified at either baseline, peak, end, or area under the curve (Fig. 3c, d). Of note, the results were similar when using either F(ab’)_2_ anti-κ + λ (Fig. 3c, d) or F(ab’)_2_ anti-IgM for BCR crosslinking (Supplementary Fig. 5a, b), as well as when analyzing CD73^-^ and CD73^+^ B_N_ cells expressing similar levels of surface IgM (Supplementary Figs. 2c and 5c, d).

In addition to low levels of surface IgM, human anergic B_N_ cells have been demonstrated to exhibit increased levels of PTEN^12^. We examined the relationship between PTEN and surface IgM expression in CD73 ^−^ and CD73^+^ B_N_ cells from 10 individuals (Supplementary Table 4) by binning IgM expressing cells as in Supplementary Fig. 2a and quantifying PTEN expression in each of the IgM-expressing B_N_ cell groups (Fig. 3e, f, Supplementary Fig. 5e). We observed that PTEN expression decreased with increasing IgM expression, as predicted. However, for B_N_ cells that expressed similar levels of IgM, the expression of CD73 was associated with a significant increase in PTEN levels (Fig. 3e, f). Thus, increased CD73 expression correlated with increased anergy in B_N_ cells_._

Taken together, the data presented here provide evidence that the major human B_N_ subset defined as IgM^low^IgD^+^ CD73^+^ that accounts for 60–70% of peripheral blood B_N_ cells are anergic. Indeed this subset expresses the anergic isotype phenotype, IgM^low^IgD^+^. Furthermore, as compared to the CD73 ^−^ B_N_ cells, CD73^+^ B_N_ cells show reduced calcium responses upon BCR crosslinking and express higher levels of PTEN associated with human B cell anergy.

### Human B cells expressing the inherently autoreactive germline V_H_4-34 are enriched in the CD73^+^ IgM^low^IgD^+^ B_N_ cell subset

Having determined that the CD73^+^ B_N_ cell subset contained the largest percent of IgM^low^IgD^+^ B cells and showed features of anergy, we analyzed the CD73^+^ and CD73 ^−^ B_N_ cell subsets for the expression of the inherently autoreactive germline V_H_4-34 heavy chain. We reasoned that if human anergic B cells were indeed autoreactive, then the CD73^+^ B_N_ cell subset would contain a larger percent of V_H_4-34 expressing B_N_ cells as compared to the CD73 ^−^ B_N_ cell subset.

**Fig. 4:**
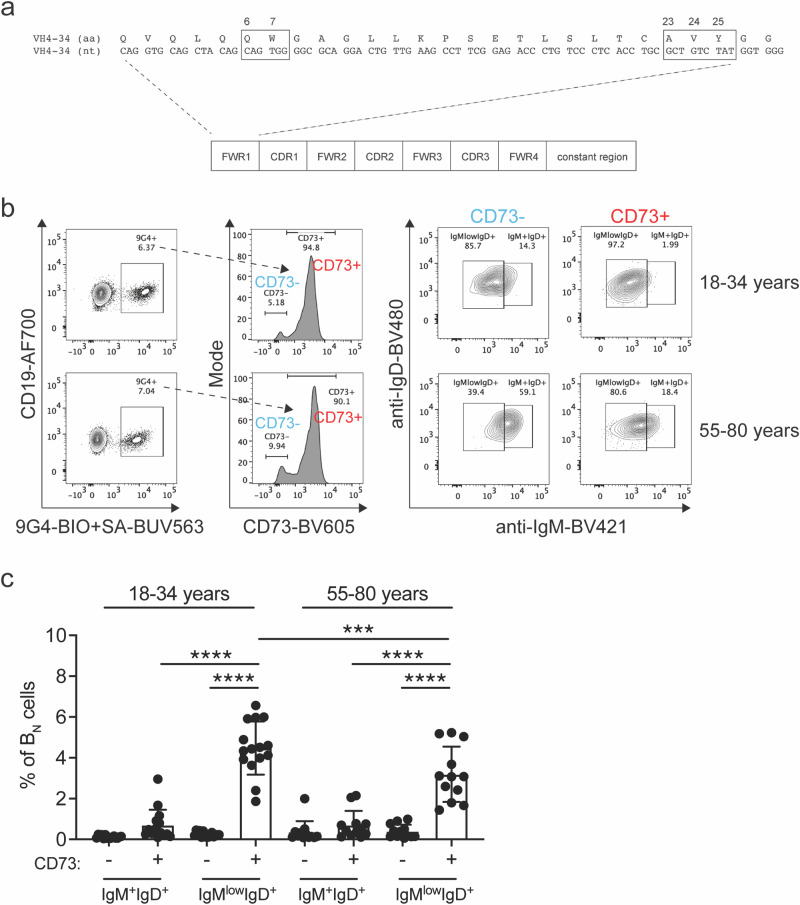
Distribution of inherently autoreactive 9G4^+^ B cells within the B_N_ cell subsets. **a** A schematic of the V_H_4-34 immunoglobulin heavy chain gene. The nucleotide and amino acid sequences of FWR1 are shown, and the hydrophobic patch is denoted in the boxes. **b** Representative flow cytometry plots from 18–34 and 55–80 year old individuals demonstrating the percentage of 9G4^+^ B cells that are CD73^-^ and CD73^+^ B_N_ cells, then further subsetted to IgM^+^IgD^+^ and IgM^low^IgD^+^ cells. **c** Summary of the percentages of 9G4^+^ cells (mean ± SD) within the B_N_ cell subsets from 15 and 12 healthy individuals in the 18–34 and 55–80 year age groups, respectively. Statistical significance was determined by one-way ANOVA test with Šidák multiple comparisons test. ***p < 0.001, ****p < 0.0001.

In humans, the expression of V_H_4-34 is a robust marker for autoreactive B cells (Fig. 4a). Antibodies utilizing germline V_H_4-34 have been demonstrated to be inherently autoreactive^20^ and the self-antigens recognized by V_H_4-34 antibodies have been well-characterized and include Ii antigen on red blood cells, as well as B cell-derived antigens including CD45^21^. In addition, critical amino acid residues in the V_H_4-34 sequence that confer the ability to bind to autoantigens have been defined^20,22,23^ and a rat-derived anti-idiotypic antibody, 9G4, that recognizes a common epitope in the FWR1 of V_H_4-34 allows the identification of V_H_4-34 expressing B cells by flow cytometry^24^.

We first determined the percent of 9G4^+^ cells within the total CD19^+^ B cells in younger and older individuals. Shown are representative flow cytometry plots for one younger and one older individual (Supplementary Fig. 6a) and quantifications of the percent of 9G4^+^ CD19^+^ B cells for 15 younger and 12 older individuals (Supplementary Fig. 6b). We observed no significant differences in the percent of total B cells that were 9G4^+^ (Supplementary Fig. 6b). We next determined within the CD73^-^ and CD73^+^ B_N_ subsets in younger versus older individuals the percent of IgM^+^IgD^+^ and IgM^low^IgD^+^ B cells that were 9G4^+^. Shown are representative flow plots for this analysis (Fig. 4b) and a quantification of these results for a large number of individuals (Fig. 4c). The CD73^+^ IgM^low^IgD^+^ anergic B_N_ cell subset contained the largest percent of 9G4^+^ cells in both younger and older individuals (Fig. 4c, Supplementary Fig. 6d). As shown in the target plots, the CD73^+^ B_N_ cell subset contained 75–80% of all CD19^+^ 9G4^+^ B cells in both younger and older individuals, while 9G4^+^ B cells were rare in the other B cell subsets, including AtBCs (Supplementary Fig. 6d). Approximately 67% of 9G4^+^ B cells expressed an anergic isotype phenotype (IgM^low^IgD^+^) in younger individuals and ~60% of 9G4^+^ B cells were anergic in older individuals (Supplementary Fig. 6d).

### Analysis of V_H_4-34 IgM sequences expressed by early plasmablasts in response to influenza vaccination showed differences in accumulation of somatic mutations between younger and older vaccines

**Fig. 5:**
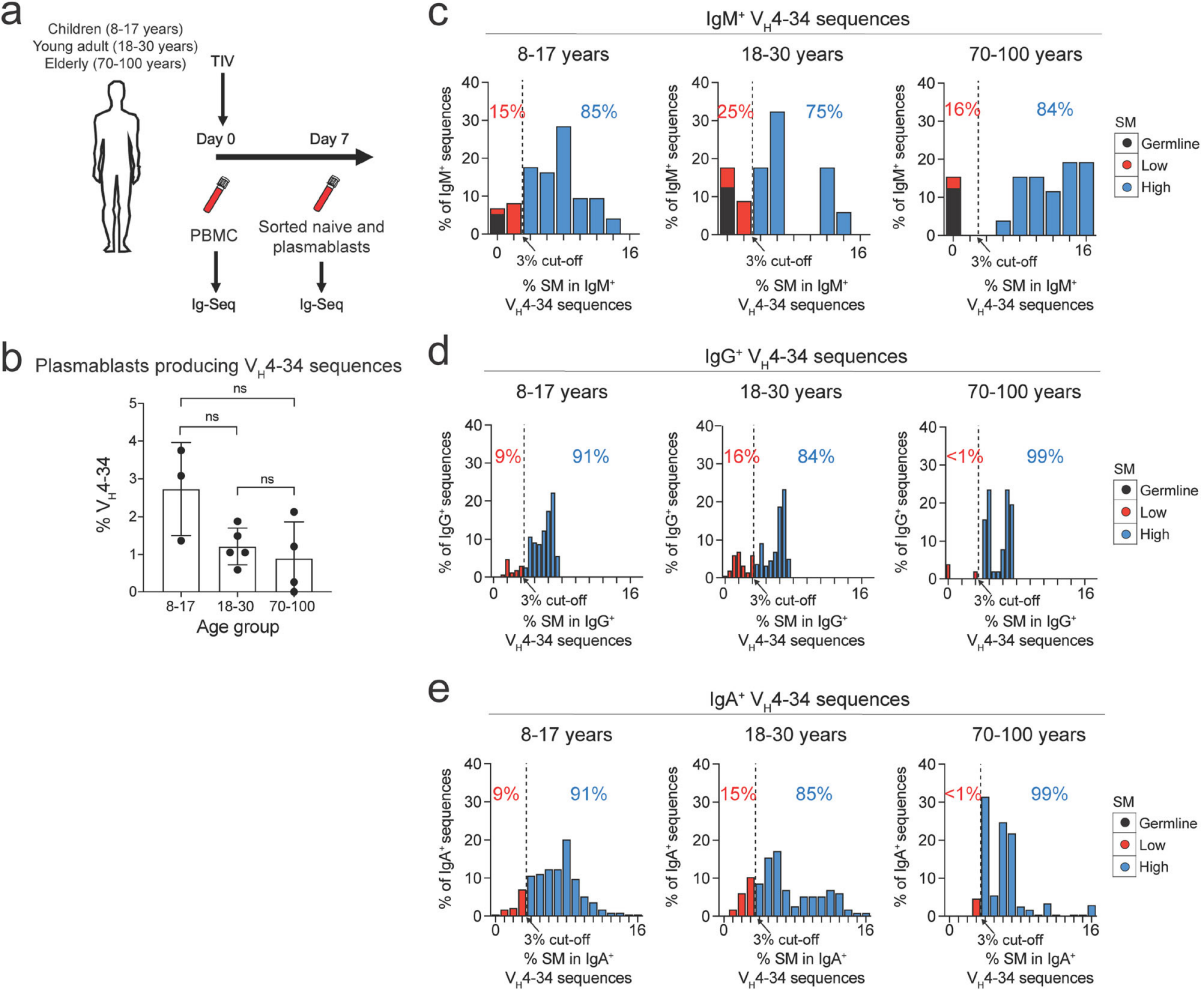
Mutation analysis of V_H_4-34 sequences produced by plasmablasts generated after influenza vaccination. **a** A scheme for the study showing the timepoints and trivalent influenzavirus (TIV) vaccination in 8–17, 18–30, and 70–100 year age groups. **b** The percentage of V_H_4-34 sequences from total plasmablast-derived sequences of all isotypes was compared between the different age groups. Statistical significance was determined by one-way ANOVA, ns= not significant. V_H_4-34 IgM^+^ (**c**), IgG^+^ (**d**), and IgA^+^ (**e**) sequences were grouped according their percent somatic mutations (% SM) and summarized in the histograms. The dotted line represents the % SM cut-off delineating SM^Low^ versus SM^High^ as shown by the arrows. The percentage of sequences that are SM^Low^ and SM^High^ are indicated in the histograms.

A critical question to address was whether anergic B_N_ cells participate in immune responses to vaccination. We carried out a sequence analysis of V_H_4-34 Ig produced by plasmablasts early (seven days) after vaccination that reflected the newly-generated plasmablast response by pre-existing B cells to vaccination. It has been observed that human B cell responses to influenza vaccination are characterized by broadly neutralizing antibodies with polyreactivity^25^. Using a publicly available data set generated to analyze the differences in the reponse to influenza vaccination due to age^26^, we analyzed the V_H_4-34 sequences expressed by sorted, purified peripheral blood plasmablasts seven days after vaccination in younger (8–17 and 18–30 years) and elderly (70–100 years) individuals^26^ (Fig. 5a). We determined that the percent of total plasmablast-derived Ig sequences of all isotypes that were V_H_4-34 were not significantly different between the vaccinated younger age groups (8–17 and 18–30 years) as compared to elderly (70–100 years) vaccinated individuals (Fig. 5b). Thus,V_H_4-34 expressing B cells were equally able to be recruited into the vaccine plasmablast responses in younger and elderly individuals.

For V_H_4-34 sequences in each age group (8–17 years; 18–30 years; 70–100 years), we determined the isotype of the sequences, IgM^+^ (Fig. 5c), IgG^+^ (Fig. 5d) or IgA^+^ (Fig. 5e) as well as the percent of somatic mutations (SM) acquired in each germline V_H_4-34 sequence. We then compared the percent mutations in V_H_4-34 sequences acquired by both groups of younger vaccinated individuals and from elderly individuals. To do so, we grouped each set of sequences as containing either low or high percentages of SMs (SM^Low^ versus SM^High^). To facilitate comparisons between age groups for each isotype, we set the cut off for SM^Low^ versus SM^High^ as the mean minus standard deviation (6.7% ± 3.7%) of one age group, namely the 70-100 year old age group (Fig. 5c). We found that the percent of SMs in the V_H_4-34 sequences was consistent with the published literature^27,28^. Our approach identified a cut-off of 3%; therefore V_H_4-34 sequences with SM >3% were termed SM^High^ (blue bars) and V_H_4-34 sequences with SM ≤3% were termed SM^Low^ (red bars). Application of the 3% cut-off revealed that 31% of V_H_4-34 sequences from PBMCs obtained prior to immunization were SM^High^, whereas 89% of V_H_4-34 sequences from plasmablasts at seven days post-vaccination were SM^High^ (Supplementary Fig. 7a). The majority of IgM^+^ sequences from both younger groups and the elderly group were SM^High^ (75–85%), whereas 15–25% of the IgM^+^ sequences were SM^Low^ (Fig. 5c, Supplementary Fig. 7b), consistent with these sequences being produced by either newly activated IgM^+^ B_N_ cells (IgM^+^ SM^Low^) or by pre-existing (IgM^+^ SM^High^) MBCs that had acquired SM in germinal centers (GCs). For IgG^+^ and IgA^+^ V_H_4-34 sequences, the vast majority in each of the age groups were SM^High^ which is consistent with the switched sequences being the product of MBCs and not B_N_ cells (Fig. 5d, e, Supplementary Fig. 7c, d).

### Following vaccination, the accumulation of SM in V_H_4-34 sequences correlated with the loss of autoreactivity

We also determined if the observed mutations in the V_H_4-34 sequences affected the ability of V_H_4-34 heavy chains to bind to autoantigens, as would be predicted of anergic B cells undergoing redemption within GCs to fill holes in the B_N_ cell repertoire. To address this question, we took advantage of the well-established observation that the three amino acid AVY patch at positions 23–25 in the germline V_H_4-34 sequence (Fig. 4a) was required for binding to a variety of self-antigens such that replacement mutations at the AVY site reduced recognition of autoantigens and likely eliminated autoreactivity^20^ We first determined whether the AVY patch was simply more prone to undergo SM. To do so, we analyzed the V_H_4-34 germline DNA sequence for the presence of “hotspots” characterized by DNA motifs targeted by activation-induced cytidine deaminase (AID) or DNA polymerase η (Polη). Overlapping AID and Polη hotspots are associated with high rates of SM^29,30^. Analysis of V_H_4-34 germline DNA sequence revealed overlapping AID and Polη hotspots in CDR regions, but no such overlap was observed at the AVY sequence (Supplementary Fig. 8a, b), providing evidence that the DNA region encoding the AVY patch was not prone to SM.

**Fig. 6:**
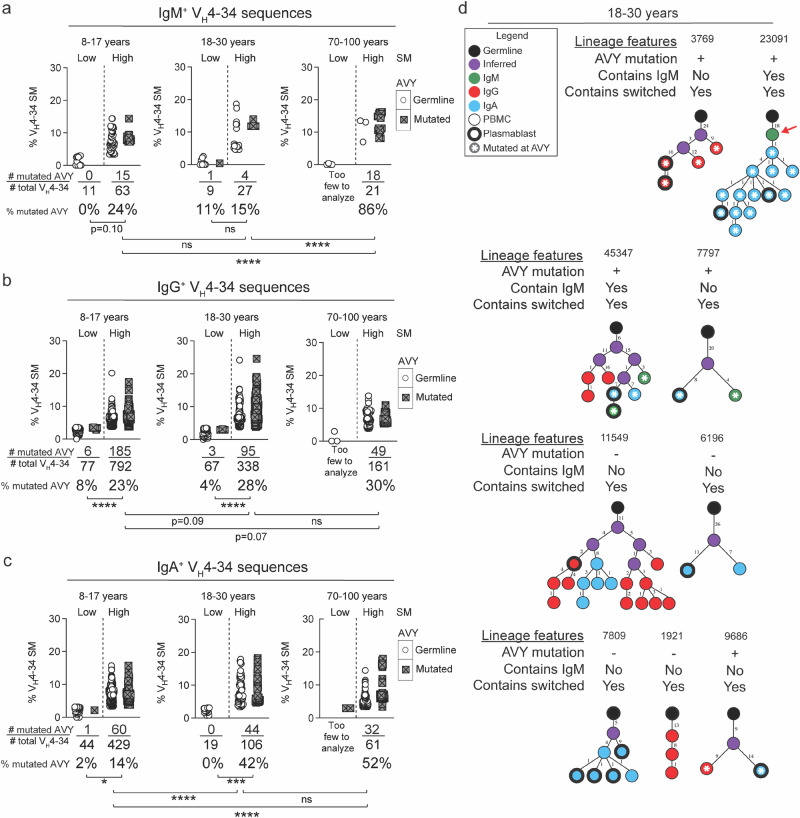
Evidence of a correlation between the accumulation of SMs in V_H_4-34 sequences by vaccination and the loss of autoreactivity. IgM+ (**a**), IgG^+^ (**b**), and IgA^+^ (**c**) V_H_4-34 sequences from plasmablasts at 7 days post-vaccination with influenza vaccine were grouped according to age (8–17, 18–30, and 70–100 years), SM^Low^ versus SM^High^, and presence/absence of AVY mutations as shown in the color-coded key. The percentage of mutated AVY sequences was analyzed by Fisher’s exact test. *p < 0.05, **p < 0.01, ***p < 0.001, ****p < 0.0001, ns=not significant. **d** Nine V_H_4-34 lineages identified by clone number from 18–30 year olds immunized with trivalent inactivated influenza vaccine are shown. The red arrow shown for lineage 23,091 highlights a B_N_ cell-derived sequence.

We next focused our analyses on V_H_4-34 sequences from the individuals immunized with influenza vaccine. In peripheral blood B cells of individuals prior to vaccination (day 0), there were negligible mutations in the AVY sequences in IgM^+^ V_H_4-34, suggesting that these V_H_4-34 sequences were derived from B_N_ cells that were not under selective pressure to mutate in the absence of an immune challenge (Supplementary Fig. 9a). For each age group of vaccinated individuals and for each V_H_4-34 isotype we determined the percent of total SM^Low^ versus SM^High^ V_H_4-34 sequences that contained a mutated AVY sequence (Fig. 6a-c). We observed a highly consistent pattern across all age groups and isotypes, namely that the SM^High^ V_H_4-34 sequences had more AVY mutations as compared to SM^Low^ sequences (Fig. 6a-c). These data suggest that the SM^High^ sequences likely had a greater loss in autoreactivity, consistent with positive selection. A similar result was obtained when comparing the percentage of AVY mutations in SM^High^ sequences between PBMCs prior to immunization and plasmablasts at seven days post-immunization, which appeared most striking in the elderly individuals (Supplementary Fig. 9b-d). Thus, for IgM^+^, IgG^+^, and IgA^+^ MBCs, the accumulation of SMs in V_H_4-34 sequences appears to correlate with the likely loss of autoreactivity.

We next determined the total number of replacement mutations and their position along IgM V_H_4-34 sequences. This analysis revealed that mutations in V_H_4-34 SM^Low^ and SM^High^ sequences were distributed throughout V_H_4-34 with no notable differences between age groups (Supplementary Fig. 10a-c). Notably, in all age groups, V_H_4-34 sequences revealed a statistically significant enrichment of replacement mutations in V_H_4-34 sequences in which the AVY sequence was mutated compared to V_H_4-34 sequences in which AVY sequence was germline, suggesting a level of antigen-driven selection (Supplementary Fig. 10a-c).

We were also able to identify clonal lineages containing AVY mutated sequences in the pre-vaccination peripheral blood B cells and in the day 7 plasmablasts for 18–30 year olds (Fig. 6d) and for 70–100 year olds (Supplementary Fig. 10d). Given are the ID numbers of clonal lineages identified in the analysis and the age group of the individuals for the lineage trees shown. Of the nine lineages in the 18–30 year old age group, five contained sequences with AVY mutations and three contained IgM^+^ sequences. The remaining six lineages in the 18–30 year old (Fig. 6d) and all five lineages in the 70–100 age group (Supplementary Fig. 10d) were dominated by IgG^+^ and IgA^+^ sequences, suggesting they were derived by pre-existing MBCs. Notably, lineage 23,091 originated from a germline IgM^+^ sequence and was clonally related to plasmablasts that had undergone mutations at the AVY patch (Fig. 6d), further suggesting that anergic B cells in younger individuals were activated in response to vaccination, mutated at the autoreactive AVY patch, and differentiated into plasmablasts. These data present direct evidence that the V_H_4-34 sequences from vaccinated young adults were undergoing mutations that would alter the ability to bind to autoantigens, thus contributing to the diversity of antibodies generated by influenza vaccination.

## Discussion

Our in-depth characterization of the human peripheral blood B_N_ cell subpopulation provided important new insights into the process of vaccination in individuals to new pathogens or their variants. Firstly, we provided several lines of evidence that the majority (60–70%) of human B_N_ cells express features of anergic human B_N_ cells. This large fraction was an unexpected finding given that the percent of B_N_ cells previously reported to be anergic was 2.5–30% as estimated by assessments of reactivity of cloned antibodies to sets of known autoantigens. However, this discrepancy could be explained at least in part by the likelihood that autoantigens assayed in prior studies did not represent the full autoantigen repertoire. Using a recently described panel of antibodies that defined all 10 human B cell subpopulations, we focused on B_N_ cells (CD19^+^CD27 ^−^ CD45RB^MEM −^ CD38^low/ −^) and confirmed that B_N_ cells could be divided into two subpopulations by the expression of CD73. Approximately 80–90% of B_N_ cells were CD73^+^, and analysis of BCR isotypes showed that the majority of these (60–70%) were of the IgM^low^IgD^+^ phenotype associated with human B cell anergy. In contrast, only 10–20% of B_N_ cells were CD73 ^−^ and among these only 5–7% were IgM^low^IgD^+^. Furthermore, we found that when compared to CD73^-^ B_N_ cells, CD73^+^ B_N_ cells exhibited reduced calcium flux upon BCR crosslinking with soluble antigen and elevated PTEN, which are features associated with B cell anergy. Thus, CD73 appears to be a distinguishing marker of human B_N_ cells expressing an anergic phenotype characterized by low levels of surface IgM. CD73 is an ectoenzyme ubiquitously expressed on many cell types in humans, mediating the extracellular conversion of AMP to adenosine^31^. It has been suggested that CD73^+^ B cells convert AMP to adenosine that mediates immunosuppressive effects on B cells^15^. Given the immunosuppressive effect of adenosine produced by CD73, it is tempting to speculate that CD73 is involved in the anergic programming of B cells. Evidence for this hypothesis includes the fact that B cell CD73 gene expression is inversely correlated with gene expression of CD48, CD82, CD79b, and IgM^14^. We observed a strong inverse relationship between the expression of CD73 and IgM on B cell surfaces, suggesting that CD73 may drive down-regulation of IgM^+^ BCR or that they are both affects of anergic B cell programming. CD73^+^ B_N_ cells express lower levels of CD86 and CD25 on their cell surface as compared to CD73 ^−^ B cells, suggesting that CD73^+^ B_N_ cells are less activated^32^. Furthermore, CD73^+^ B_N_ cells expressed higher levels of BAFF-R but not TACI than CD73 ^−^ B_N_ cells, suggesting a higher requirement for survival signals^32^. An exploration of mechanisms that might underlie a potential link between CD73 and B_N_ cell responses were beyond the scope of our study. However, it will be of considerable interest to explore a possible link between the function of CD73 and the anergic state of CD73^+^ B_N_ cells in future studies.

An analysis of the expression of the inherently autoreactive V_H_4-34 heavy chain identified by the 9G4 anti-idiotype provided critical evidence that the majority of autoreactive V_H_4-34 expressing cells in human peripheral blood were contained in the CD73^+^ IgM^low^IgD^+^ B_N_ subset. Indeed, approximately 5% of CD73^+^ B_N_ cells expressed autoreactive V_H_4-34^+^ BCRs that accounted for nearly all (~80%) of 9G4^+^ B cells in human peripheral blood. The observation that human B_N_ cells show evidence of anergy strongly suggests that they are not antigen-inexperienced, but rather that they have experienced self-antigens that were presumably responsible for their anergic state.

We also provided crucial evidence that 9G4^+^ anergic B cells responded to vaccination. To do so, we analyzed the IgM^+^ V_H_4-34 sequences in peripheral blood in individuals before influenza vaccination and sequences within sorted plasmablasts seven days after vaccination. We identified both germline V_H_4-34 sequences and V_H_4-34 sequences that had acquired SMs. We provided evidence that in younger vaccinated individuals that approximately half of IgM^+^ V_H_4-34 sequences were either germline or had accumulated only a small number of SMs. Importantly these sequences had remained autoreactive through maintenance of their germline AVY sequences, suggesting that these sequences were likely produced in B_N_ cells recently activated by vaccination. In contrast, approximately half of IgM^+^ V_H_4-34 sequences had acquired a large number of mutations and had lost their autoreactivity through mutation of their AVY sequences, suggesting that these were from pre-existing IgM^+^ MBCs derived from anergic B_N_ cells that had undergone additional diversification and mutation away from autoreactivity. Taken together, these findings suggest that in vaccinated younger individuals, a diverse repertoire of vaccine-responsive B cells is acquired by the recruitment of highly mutated pre-existing MBCs which eliminated their autoreactivity as well as recently activated anergic B_N_ cells expressing autoreactive BCRs with low levels of SMs.

In contrast to the younger individuals, in elderly individuals (70–100 years), the vast majority of IgM^+^ V_H_4-34 sequences were highly mutated, indicating that these may have been produced by pre-existing IgM^+^ MBCs rather than from B_N_ cells. There were almost no germline IgM^+^ V_H_4-34 sequences or sequences that had low levels of SM in elderly individuals, suggesting the plasmablasts that produced these were from pre-existing IgM^+^ MBCs that had undergone additional SMs rather than B_N_ cells. These observations are consistent with the findings of Jiang et al.^26^, who generated and analyzed the clonal structure and mutational distribution of individual repertoires of the Ig sequences analyzed here. They showed that elderly individuals had a decreased number of lineages but an increased prevaccination mutational load in their repertoire. In addition, some individuals had an oligoclonal nature to their repertoire in which the diversity of the lineages was greatly reduced as compared to that in younger individuals. Our analysis of these same V_H_4-34 sequences offers a possible explanation for their observations. If the germline IgM^+^ V_H_4-34 sequences and sequences with small numbers of mutations were derived from vaccine-activated anergic B_N_ cells, the paucity of such V_H_4-34 sequences in the elderly suggest that the elderly have a diminished capacity to acquire new mutations in their anergic B_N_ cells, and instead rely on pre-existing mutated IgM^+^ MBCs that may limit their ability to respond to new pathogen variants such as those that occur in influenzavirus infections. Our conclusion is consistent with those of investigators who compared antibody responses to influenza vaccination in young and elderly individuals and attributed observed differences to defective GC responses in the elderly^33–35^.

We identified V_H_4-34 lineages providing evidence for the stepwise process that ultimately resulted in the production of vaccine-induced antibodies. Our findings are consistent with the ‘clonal redemption’ hypothesis put forward by Goodnow and colleagues that offered an explanation for how autoreactive anergic B cells can be repurposed to contribute to pathogen-specific immune responses^8^ and to fill holes in the B cell repertoire. The basic tenet of the model is that self-reactive anergic B cells are ‘awakened’ and recruited into GCs where they somatically mutate away from self reactivity allowing diversification of the naive repertoire and positive selection by foreign antigens^8,10^. It is also of interest that IgM^+^ V_H_4-34 SM^High^ sequences in the elderly showed the largest percent of AVY mutations (85%) and consequently greatest loss of autoreactivity as compared to younger individuals (42%). Autoreactivity has been suggested to play an important role in increasing the avidity of bivalent antibodies for pathogen cell surface expressed epitopes^36^. The loss of self-reactivity could contribute to less effective responses to vaccination in the elderly.

The findings presented here highlight the importance of understanding the mechanisms by which anergic B_N_ cells existing in a quiescent state could be activated to participate in a GC reaction. The studies presented here identify B_N_ cells that express an isotype phenotype, IgM^low^IgD^+^, that has consistently been shown to be associated with an anergic or hyporesponsive state^6,7,12,24,37^. This definition allowed us to assess the entire B_N_ cell subset for anergy without knowing the B_N_ cells’ antigen specificities or requirements for antigen-driven activation. Although this global view provided several important insights, it will be necessary in future studies to understand the requirements for activation of anergic human B_N_ cells of known antigen specificities and known autoreactivities. It is reasonable to anticipate that there may be considerable heterogeneity both in the details of the mechanisms regulating the anergic state as well as in the requirement for awakening from anergy. We previously showed that B cells within the human AtBC subset, an IFN-γ-dependent lineage that is expanded in chronic infectious diseases and in autoimmunity, were hyporesponsive to soluble antigen^17^ and were described as ‘exhausted’^38^. However, these cells responded robustly to membrane-associated antigens^17,37^. We provided evidence that this unique activation requirement correlated with a high activation threshold of these B cells mediated by the inhibitory receptor FcγRIIB^37^, possibly to avoid awakening by soluble self-antigens but to promote full activation by antigens presented on membranes. Taken together, these findings suggest the interesting possibility that in B cells anergy functions to regulate threshold for activation of autoreactive B cells so that they are ‘awakened’ only under conditions in which they can subsequently initiate the process of clonal redemption.

An important implication of our findings is that highly successful vaccines will require the ability to awaken anergic B cells. In this regard, it is of genuine interest that the current highly effective human papillomavirus (HPV) virus-like particle (VLP) vaccine has been demonstrated in mouse models to overcome anergy allowing for an antibody response to self antigens^39,40^. An understanding of the mechanisms by which HPV-VLP vaccines are able to break anergy should be highly beneficial to development of effective vaccines for pathogens for which we have none.

Taken together, along with our recent description of differences in affinity thresholds for activation of IgM^+^ versus IgG^+^ MBCs^41^, we favor a model in which human anergic B_N_ cells are awakened and activated by vaccination to begin SM, ultimately entering GCs where further SM, class switching, selection, and differentiation occur, resulting in the production of a highly selected IgG^+^/IgA^+^ MBC repertoire and a weakly selected broad IgM^+^ MBC repertoire. Upon homologous pathogen challenge, IgG^+^ MBCs are activated to differentiate to plasma cells, providing protection against the homologous pathogen. IgM^+^ MBCs are able to respond to low affinity heterologous pathogen challenge and are activated to enter GC and produce IgG^+^ MBCs highly selected for reactivity to the heterologous pathogen as well as replenish the broad IgM^+^ MBC population. Our data suggest that, in younger individuals, anergic B_N_ cells are activated to generate highly selected and highly mutated switched MBCs as well as IgM^+^ MBCs, which can be activated by subsequent antigen challenges that generate highly selected plasma cells. In contrast, in older individuals, responses to subsequent antigen challenges rely on pre-existing IgM^+^ MBCs to generate highly selected plasma cells.

In summary, we provided evidence that the circulating CD19^+^ B_N_ pool in peripheral blood in humans is heterogeneous but dominated by B cells of an anergic naive phenotype and marked by expression of CD73. Further understanding the functional properties of these CD73^+^ IgM^low^IgD^+^ B_N_ cells may provide valuable information on how to harness this pool to improve vaccine responses as well as control responses in the context of autoimmunity.

## Methods

### Healthy human subjects, study approval, and isolation of peripheral blood mononuclear cells (PBMCs)

The de-identified blood samples were obtained from NIH protocol # 99-CC-0168 “Collection and Distribution of Blood Components from Healthy Donors for In Vitro Research Use,” which was approved by the NIH Intramural Research Program IRB committee and in accordance with the Declaration of Helsinki. Donors meeting research donor eligibility criteria were recruited to donate blood and blood components by standard phlebotomy and apheresis techniques. The investigational nature of the studies in which their blood will be used and the risks of the donation process were carefully explained to the donors, and a signed informed consent document was obtained. Whole blood samples from healthy U.S. adult donors were obtained, and PBMCs were isolated within six hours after the blood draw and EDTA was used as the anticoagulant. The demographics of the individuals analyzed in this study are included in Supplementary Tables 1-4. Sex was not considered a biological variable. Race was self-reported by the participants. The de-identified blood samples were centrifuged at 1500 rpm for 15 minutes to separate the plasma from the cellular fraction, which contains the PBMCs. The PBMCs were then resuspended in sterile PBS and layered over Ficoll-Paque PLUS (Cat#17144002, Cytiva). After centrifugation at 500 g at 10 minutes with minimal acceleration/deceleration, the PBMCs were isolated, washed extensively with sterile PBS (Cat#21-040-CV, Corning), and counted. The PBMCs were then resuspended in sterile freezing medium comprised of heat-inactivated fetal calf serum (Cat#SH30088.03, Cytiva) with 10% DMSO (Cat#2650-5X5ML, Sigma) and stored in liquid nitrogen for later use.

### Flow cytometry of human B cells from peripheral blood

B cell subpopulations from PBMCs were identified by antibodies against CD19 (clone SJ25-C1, Cat#9340-27, Southern Biotech), CD11c (clone B-ly6, Cat#741827, BD Biosciences), CD27 (clone O323, Cat#302832, Biolegend), CD45RB^MEM^ (clone MEM-55, Cat#310204, Biolegend), CD38 (clone HIT2, Cat#563965, Biolegend), CD73 (clone AD2, Cat#344024, Biolegend), and CD95 (clone DX2, Cat#305634, Biolegend) as previously described^14^. Dead cells were excluded by use of Live/dead near IR (Cat#L34976, Thermo Fisher). Isotype expression was measured with monoclonal antibodies against IgM (clone MHM-88, Cat#314534, Biolegend), IgG (clone G18-145, Cat#564229, BD Biosciences), IgD (clone IA6-2, Cat#566138, BD Biosciences), and IgA (clone IS11-8E10, Cat#130-113-477, Miltenyi Biotec). B cells that were negative for these isotype-specific antibodies were termed ‘Other’ but assumed to express IgE. Autoreactive B cells were identified with biotinylated 9G4 (rat IgG2aκ, a generous gift from Dr Iñaki Sanz at Emory University), which recognizes BCRs utilizing germline V_H_4-34^9^, with streptavidin-BUV563 (Cat#612935, BD Biosciences) for secondary detection. All surface marker staining was performed on ice with Brilliant Buffer PLUS (Cat#566385, BD Biosciences) and Fc block (Cat#564219, BD Biosciences). For intracellular staining, cells were then fixed and permeabilized with BD Transcrption Factor Kit (Cat#562574, BD Biosciences) according to the manufacturer’s instructions. Permeabilized cells were stained with antibodies against PTEN (Cat#IV847G-100UG, R&D Systems) or isotype control (clone X40, Cat#567121, BD Biosciences; clone MOPC-21, cat#557783, BD Biiosciences). After staining, prepared samples were analyzed by flow cytometry using a BD FACSymphony A5 after compensation with UltraComp eBeads^TM^ compensation beads (cat#01-2222-42, Invitrogen). The data were analyzed using FlowJo version 10.9.0 and Prism version 10.0.2 software.

### Calcium flux assay

B_N_ cells from peripheral blood of participants described in Supplementary Table 3 were first purified with EasySepTM Human Naive B Cell Isolation Kit (cat#17254, StemCell^TM^ Technologies) according to manufacturer’s instructions, and purified cells were stained with labeled antibodies as previously described with the notable exceptions: surface IgM was labeled with goat polyclonal Fab anti-human IgM (cat#109-587-043, Jackson ImmunoResearch), surface IgD was labeled goat polyclonal Fab anti-human IgD (custom production with SouthernBiotech), and anti-CD73 (clone 4G4, cat#HM2215, HycultBiotech) custom conjugated to AlexaFluor-647 according to manufacturer’s instructions (cat#20186, ThermoFisher Scientific). Post-sort purity was measured by flow cytometry to detect live CD19^+^CD27^-^CD45RB^MEM-^ B_N_ cells, and was consistently >97%. The cells were rested for two hours at 37 °C prior to loading the cells with the calcium dye Cal-520 (cat#ab171868, abcam) in 1 mM probenecid (cat#P36400, ThermoFisher Scientific) in RPMI without phenol red (cat#11835030, ThermoFisher Scientific) at a concentration of 5 × 10^6^ cells/mL. For calcium flux, the dye-loaded cells were suspended at 5 × 10^6^ cells in 500μL 1 mM probenecid in RPMI without phenol red. Baseline readings were obtained for 30 seconds, followed by addition of F(ab’)2 anti-κ+λ (cat#2062-01 and cat#2072-01, both from SouthernBiotech) or F(ab’)2 anti-μ (cat#2022-01, SouthernBiotech) at 5μg/mL or 10μg/mL. Calcium flux responses were measured for a total of five minutes. The data were analyzed using FlowJo version 10.9.0 and Prism version 10.0.2 software.

### B cell repertoire analysis

Immunoglobulin sequences from human subjects that received an influenza vaccine were previously described and are publicly available for further analysis at Sequence Read Archive, accession ID SRX190717^26^. For processing and alignment of raw sequences, pRESTO was used following the vignette (https://presto.readthedocs.io/en/stable/workflows/Jiang2013_Workflow.html) that is tailored to this dataset. For mutation analysis and lineage reconstruction, standard workflow from the Immcantation Framework were used as described under ‘B cell repertoire analysis’. Productive V_H_4-34 sequences from 3 children (8–17 years old), 5 young adults (18–30 years old), and 4 elderly adults (70–100 years old) at days 0 and 7 were analyzed for percentage of mutations, isotype, and the presence of replacement mutations at codon positions 23–25. Mutation load in IGHV4-34 sequences was calculated using observedMutations function from the Immcantation framework package SHazaM, with replacement or substitution mutation frequencies reported as indicated across either the whole sequence, individual codons, or IMGT framework (FWR) or complementarity determining region (CDR) annotations^42,43^. To capture changes in clonally related sequences across pre-vaccination peripheral blood B cells and day 7 plasmablasts, sequences from both these visits were merged into a single .fasta file for each donor, and clonal assignment was performed using sequence similarity thresholds based on SHazaM’s distToNearest function and nucleotide Hamming Distance model. Lineage reconstruction of IGHV4-34 sequences was done with buildPhylipLineage function from the Alakazam package^44,45^, which uses the dnapars tool from PHYLIP^46^. Custom code used for mutation analysis and phylogenetic lineage reconstruction are available at https://github.com/rpholla/Influenza-vaccination-B-cell-responses.git.

### Quantification and statistical analysis

All flow cytometry data were analyzed using FlowJo and Prism software. For comparisons of flow cytometry data between 18–34 and 55–80 year old healthy individuals, including B cell subpopulations, subsets, isotypes, 9G4 binding, and percentage of CD73^+^ B_N_ cells within a given relative surface IgM level, statistical significance was determined by Mann-Whitney test, and a *p*-value < 0.05 was considered to be significant. For surface IgM levels between CD73^-^ and CD73^+^ B_N_ cells in younger and older individuals, statistical significance was determined by Kruskal-Wallis test, and *p* < 0.05 was considered statistically significant. For quantification of CD73 versus surface IgM expression, PTEN versus surface IgM expression, and calcium flux responses between CD73^-^ and CD73^+^ B_N_ cells, statistical significance was determined by paired Wilcoxon test, and a *p*-value < 0.05 was considered significant. For comparisons of four B_N_ cell subsets determined by CD73 and IgM expression, statistical significance was determined by one-way ANOVA test with Šidák multiple comparisons test, and a *p*-value < 0.05 was considered significant. For mutation analyses, statistical analyses were performed on Prism software and RStudio. For comparisons of each BCR isotype, pooled SM^Low^ versus SM^High^ V_H_4-34 sequences from three participants in the 8–17 year group, five in the 18–30 year group, and four in the 70–100 year old group. Statistical significance was determined by Fisher’s exact test, and a *p* < 0.05 was considered statistically significant. For mutation analyses at each codon position between germline AVY and mutated AVY V_H_4-34 sequences, V_H_4-34 sequences were pooled according to isotype (IgM^+^, IgG^+^, and IgA^+^) and age group, and the percent mutation at each codon position was calculated. Statistical significance was determined by Fisher’s exact test, and a *p* < 0.05 was considered statistically significant.

## Supplementary information

Supplementary information

## Data Availability

The flow cytometry files used to generate the data in Figs. 1–4 and Supplemental Figs. 1–6 are available upon request to the corresponding authors. The datasets used to generate the data analyzed in Figs. 5–6 and Supplemental Figs. 7-10 of the current study are publicly available (NCBI SRA accession ID: SRX190717, https://www.ncbi.nlm.nih.gov/sra/?term=SRX190717; GenBank AJ564425.1). Processed files in the Adaptive Immune Receptor Repertoire (AIRR) format are available for download at https://github.com/rpholla/Influenza-vaccination-B-cell-responses.git.

## Code availability

The underlying code used to generate data for this study is available at https://github.com/rpholla/Influenza-vaccination-B-cell-responses.git. RStudio Version 2023.06.2 + 561, Excel Version 16.94, and GraphPad Prism Version 10.2.0. Any additional information required to reanalyze the data reported in this paper is available from the lead contact upon request.
