# Supplementary material for: Human naïve B cells show evidence of anergy and clonal redemption following vaccination
Source: NPJ Vaccines. 2025 May 14;10:96. doi: 10.1038/s41541-025-01133-w (PMC12078529; doi:10.1038/s41541-025-01133-w)
Supplement: Supplementary file 1 — Supplementary information [file 41541_2025_1133_MOESM1_ESM.pdf]

Human naïve B cells show evidence of anergy and clonal redemption following vaccination

Brian LP Dizon[1,2,*], Prasida Holla[3], Evan Mutic[2], Paul Schaughency[4], and Susan K Pierce[2,*]

*To whom correspondence may be addressed. Brian LP Dizon: 5625 Fishers Lane, Room 4S04B, Rockville, MD, USA 20852; phone (240)651-6803. Susan K Pierce: 5625 Fishers Lane, Room 4S04E, MSC 9418, Rockville, MD, USA 20852; phone (301)980-6362.

Email: brian.dizon@nih.gov, spierce@nih.gov

This file includes:

- Supporting text
- Supplementary Figures 1 to 10
- Supplementary Tables 1 to 4

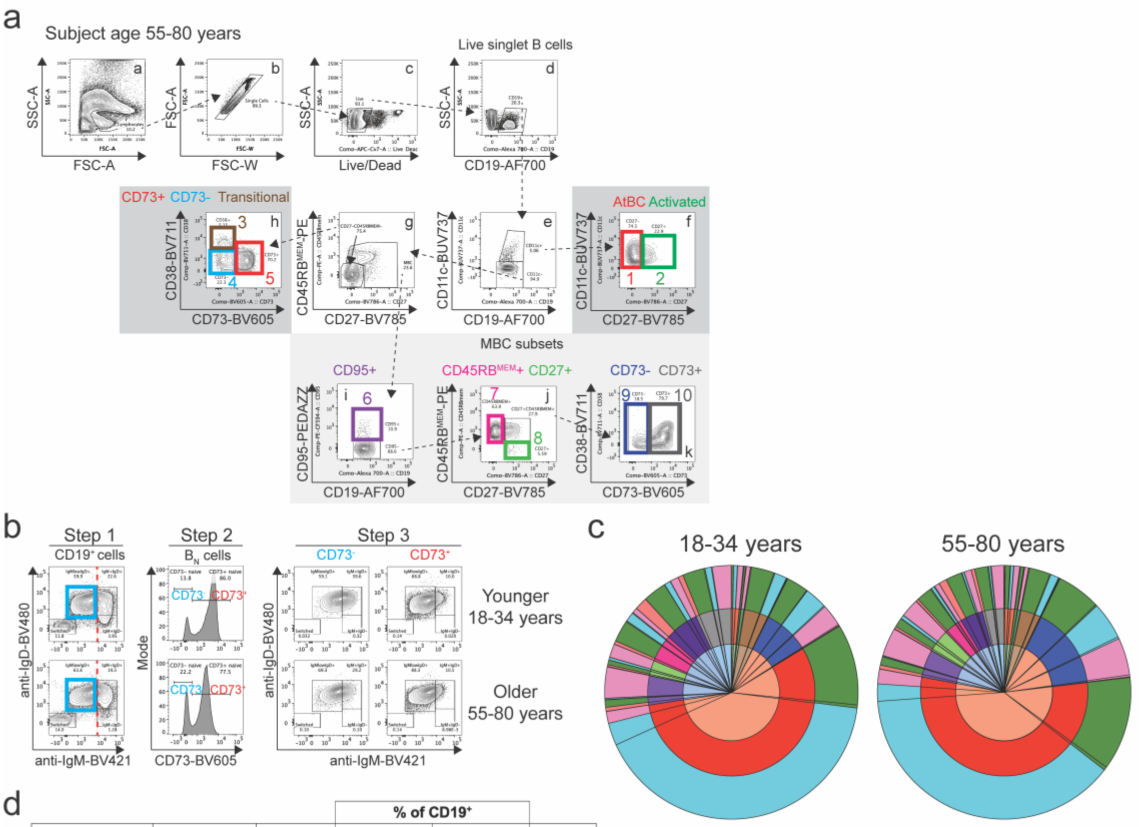

d

Subpopulation	Subset	Subset #	% of CD19⁺ 18-34 years	% of CD19⁺ 55-80 years	p-value
CD11c⁺			2.5 ± 1.3	3.3 ± 2.3	ns
	AtBC	1	1.6 ± 0.8	2.1 ± 1.4	ns
	Activated	2	0.9 ± 0.6	1.1 ± 1.0	ns
Transitional		3	4.8 ± 2.4	4.1 ± 1.3	ns
Naïve			63 ± 13	66 ± 12	ns
	CD73⁻	4	6.4 ± 2.9	12 ± 3.3	***
	CD73⁺	5	55 ± 11	55 ± 12	ns
MBC			30 ± 13	25 ± 11	ns
	CD95⁺	6	5.8 ± 2.8	5.5 ± 1.9	ns
	CD45RB^{MEM+}	7	5.3 ± 2.4	3.7 ± 1.9	*
	CD27⁺	8	3.0 ± 1.9	4.5 ± 4.9	ns
	CD73⁻	9	6.7 ± 3.9	4.6 ± 2.3	ns
	CD73⁺	10	7.3 ± 4.3	4.5 ± 3.2	0.06

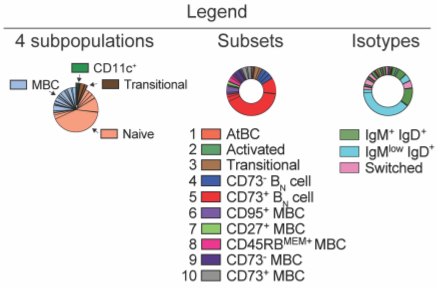

Supplementary Figure 1. Visualizing the distribution of the known peripheral blood B cell subsets according to cell surface markers and isotype, related to Figure 1.

a. The 10 B cell subsets in peripheral blood are color-coded and numbered in the flow cytometry gating strategy for a representative 55-80 year old human subject in the study.

b. Representative flow cytometry plots to identify the four peripheral blood B cell subsets distinguished by IgM and IgD. In Step 1, four populations of B cells are discriminated by isotype expression, which include $IgM^{+}IgD^{+}$, $IgM^{low}IgD^{+}$ (indicated by blue box), $IgM^{+}IgD^{-}$, and $IgM^{-}IgD^{-}$ (switched). The red dotted line indicates the separation between IgM^{low} and IgM^{+} B cells. In Step 2, $CD73^{-}$ and $CD73^{+}$ B_N cells are identified. In Step 3, the gates created in Step 1 are applied to $CD73^{-}$ and $CD73^{+}$ B_N cells.

c. A summary of the B cell populations from 18-34 and 55-80 year old individuals is shown in the target plots. The plots depict the four subpopulations (innermost), followed by the 10 subsets, then isotypes (outermost). B cells not expressing IgM or IgD are referred to as switched.

d. The percentages of the four subpopulations and 10 subsets, including $CD73^{-}$ and $CD73^{+}$ naïve B (B_N) cells, among total $CD19^{+}$ cells are shown in the table as mean ± SD from 15 individuals in the 18-34 years and 12 subjects in the 55-80 year old groups. Statistical significance was determined by Mann-Whitney test, and a p-value <0.05 was considered significant. *p<0.05, *** p<0.001, ns= not significant.

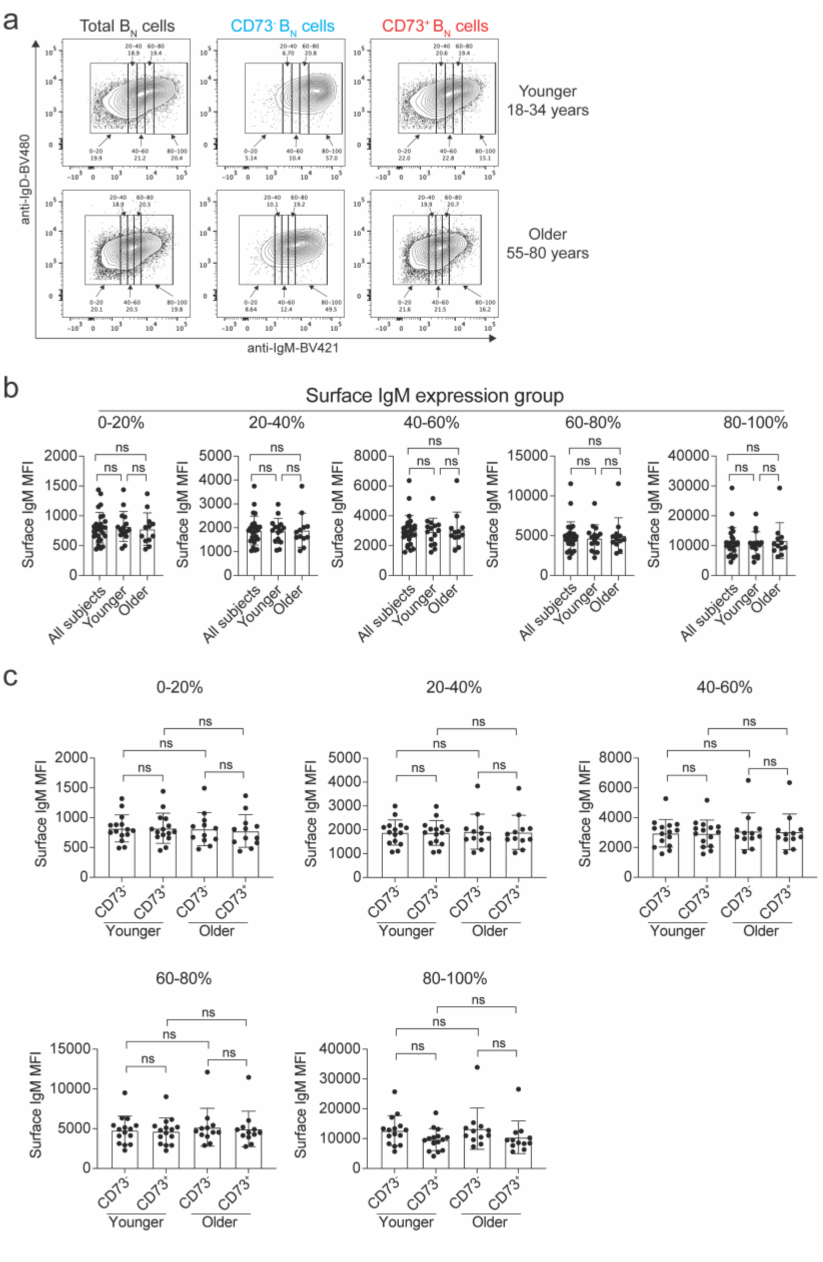

Supplementary Figure 2. Flow cytometry analysis of naïve B (B_N) cells across a continuum of surface IgM expression, related to Figure 3.

a. Representative gating of B_N cells from a younger and older individual into five groups according to relative levels of surface IgM expression (0-20%, 20-40%, 40-60%, 60-80%, and 80-100%). The gates were then applied to $CD73^-$ B_N and $CD73^+$ B_N cells.

b. Summary of surface IgM staining (mean MFI ± SD) in all subjects (younger and older) versus younger (n=12) and older (n=15). The data were analyzed by Kruskal-Wallis test for multiple comparisons, and p<0.05 was considered statistically significant. ns= not significant.

c. Summary of surface IgM staining (mean MFI ± SD) in $CD73^-$ and $CD73^+$ B_N cells in younger (n=12) and older (n=15) individuals. The data were analyzed by Kruskal-Wallis test for multiple comparisons, and p<0.05 was considered statistically significant. ns= not significant.

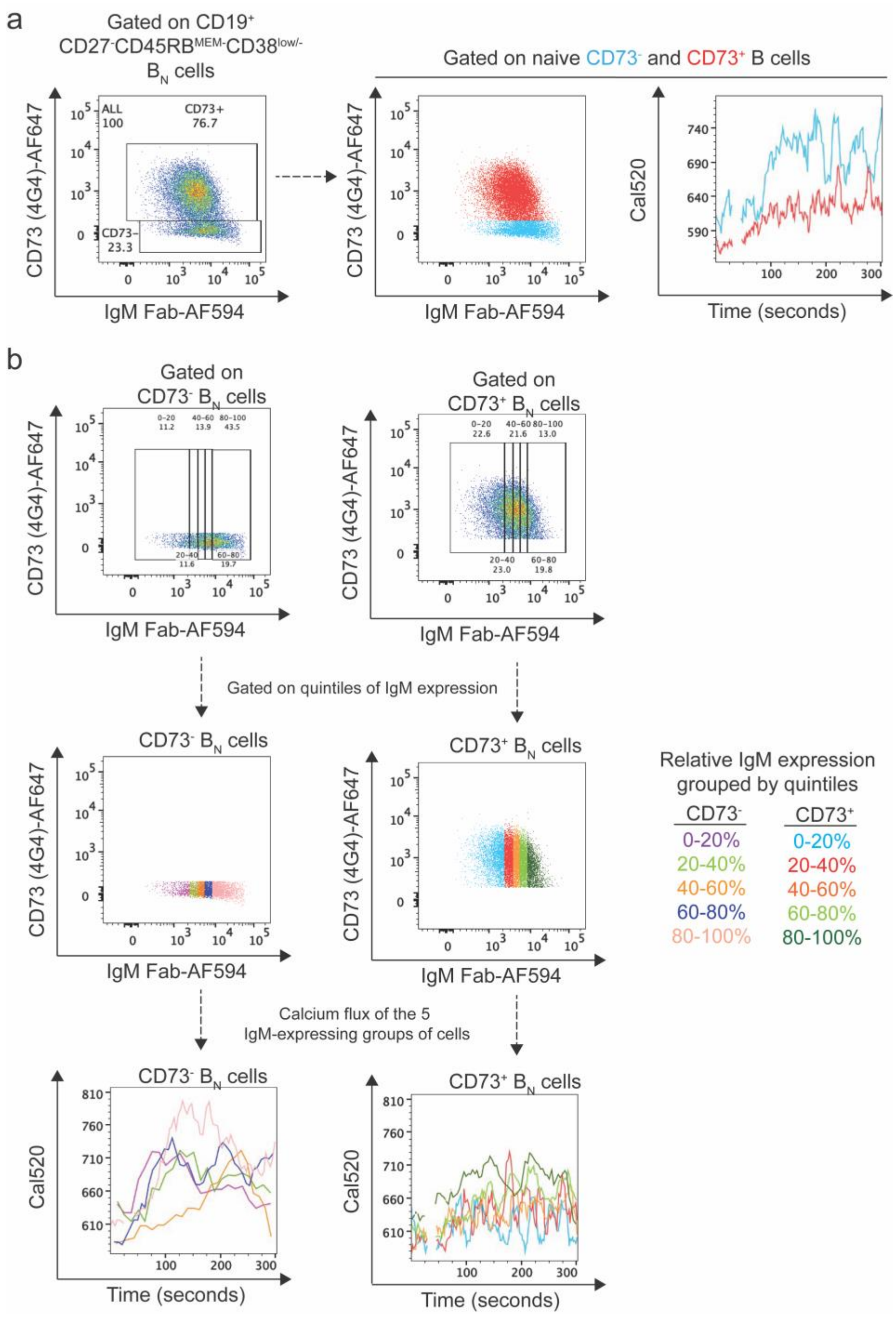

Supplementary Figure 3. Relationship between CD73 and IgM expression on B_N cell activation, related to Figure 3. B_N cells were purified with EASYSEP human naïve B cell kit to >95%, stained with CD27, $CD45RB^{MEM}$, CD38, and CD73 (clone 4G4), as well as IgD Fab-DyLight405 and IgM Fab-AF594, loaded with the calcium dye Cal520, then activated with 10μg/mL of F(ab')2 anti-κ+λ.

a. The calcium fluxes of $CD73^-$ and $CD73^+$ B_N cells are shown.

b. $CD73^-$ and $CD73^+$ B_N cells were divided into five quartiles based on relative IgM expression to define 10 populations of B_N cells and color-coded. The calcium flux responses to 10μg/mL of F(ab')2 anti-κ+λ of these 10 populations were compared.

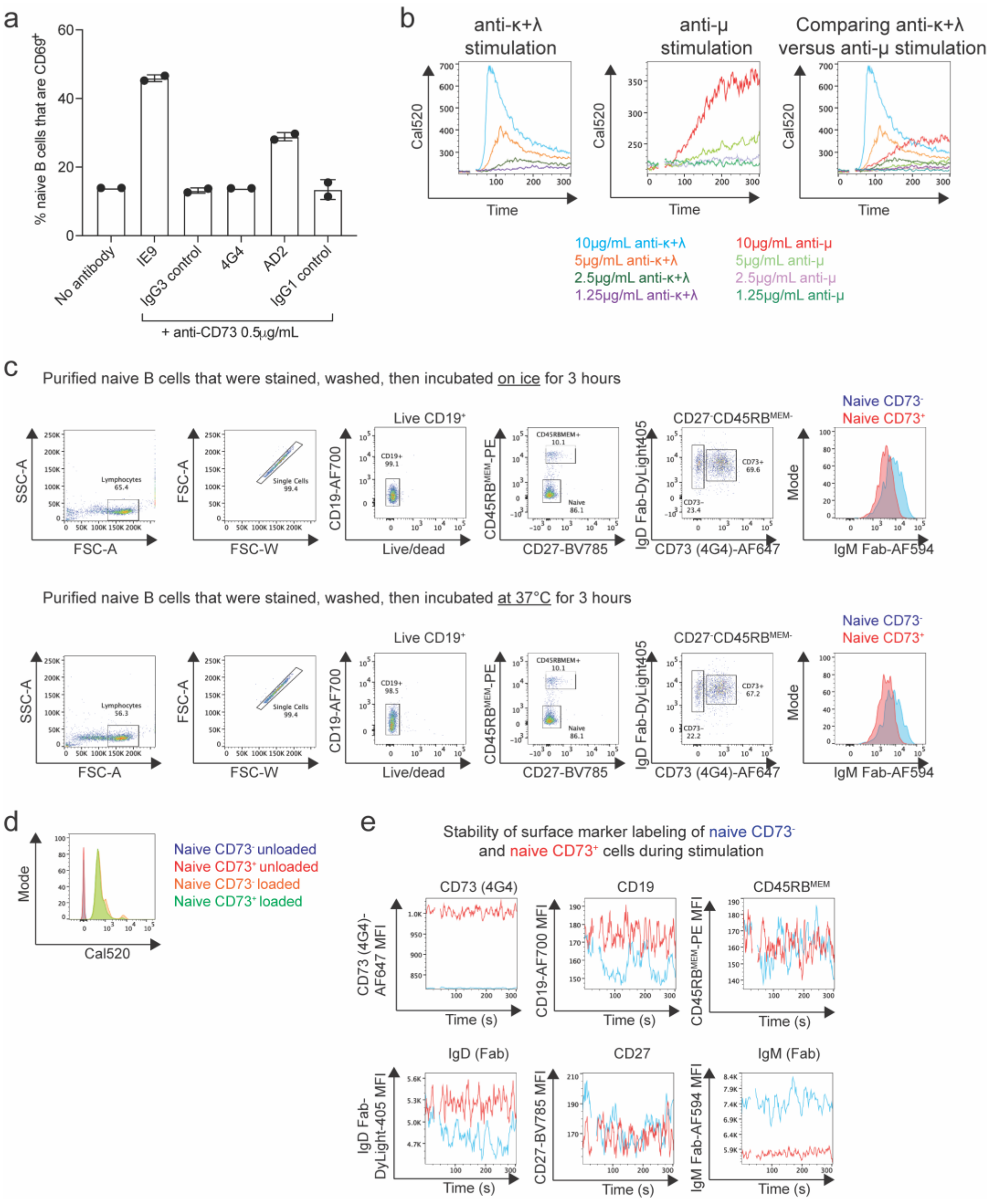

Supplementary Figure 4. Comparing B cell activation between CD73$^-$ versus CD73$^+$ B$_N$ cells, related to Figure 3.

a. Analysis of B activation as measured by CD69 expression of purified B$_N$ cells cultured for 18 hours with anti-CD73 antibodies IE9, 4G4, and AD2 versus isotype control. Clone 4G4 was used for all subsequent calcium flux studies.

b. Calcium flux to doses of soluble F(ab')2 anti-κ+λ versus F(ab')2 anti-μ of purified B$_N$ cells from peripheral blood.

c. Comparison of surface marker phenotype and flow cytometry gating strategy of purified naïve B cells that were either stained on ice for 3 hours versus at 37°C for 3 hours.

d. Comparison of Cal520 loaded between CD73$^-$ and CD73$^+$ B$_N$ cells.

e. Comparison of labeled surface marker labeling between CD73$^-$ and CD73$^+$ B$_N$ cells during B cell receptor stimulation for the first 300 seconds.

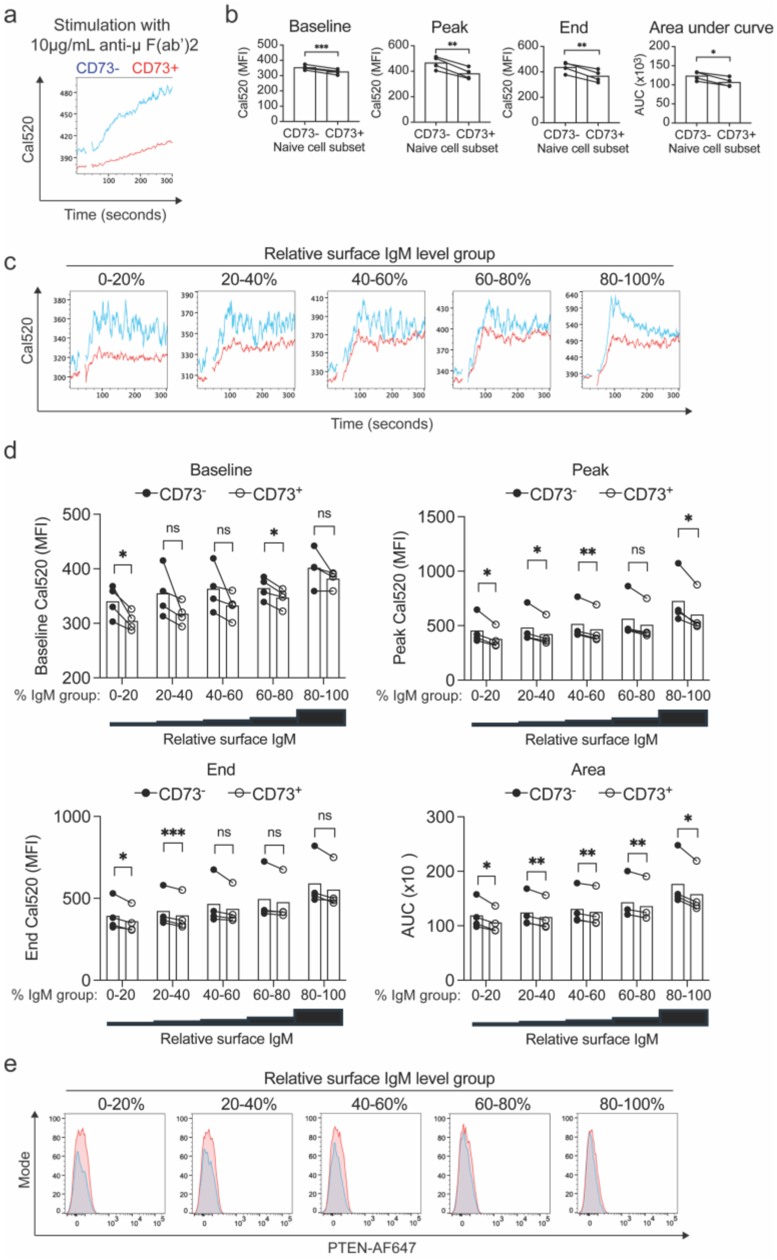

Supplementary Figure 5. Analysis of anergy by calcium flux responses of CD73⁻ and CD73⁺ B_N cells to stimulation with F(ab')2 anti-μ and intracellular PTEN, related to Figure 3.

a-b. Representative flow cytometry plot of calcium flux responses of $CD73^-$ and $CD73^+$ B_N cells to 10μg/mL F(ab')2 anti-μ (a), and summaries of the baseline, peak, and calcium flux responses in four unrelated individuals (b). Statistical significance was determined by paired Wilcoxon test, and p-value <0.05 was considered significant. * p<0.05, **p<0.01, and *** p<0.001.

c-d. Representative flow cytometry plot of calcium flux responses of $CD73^-$ and $CD73^+$ B_N cells to 10μg/mL F(ab')2 anti-κ+λ (c). The cells were divided according to relative IgM levels as in Supplemental Figure S2, and summaries of the baseline, peak, and calcium flux responses in four unrelated individuals (d). Statistical significance was determined by paired Wilcoxon test, and p-value <0.05 was considered significant. * p<0.05, **p<0.01, and *** p<0.001, ns= not significant.

e. Representative flow cytometry plots showing intracellular PTEN staining according to relative surface IgM grouping in $CD73^+$ versus $CD73^-$ B_N cells.

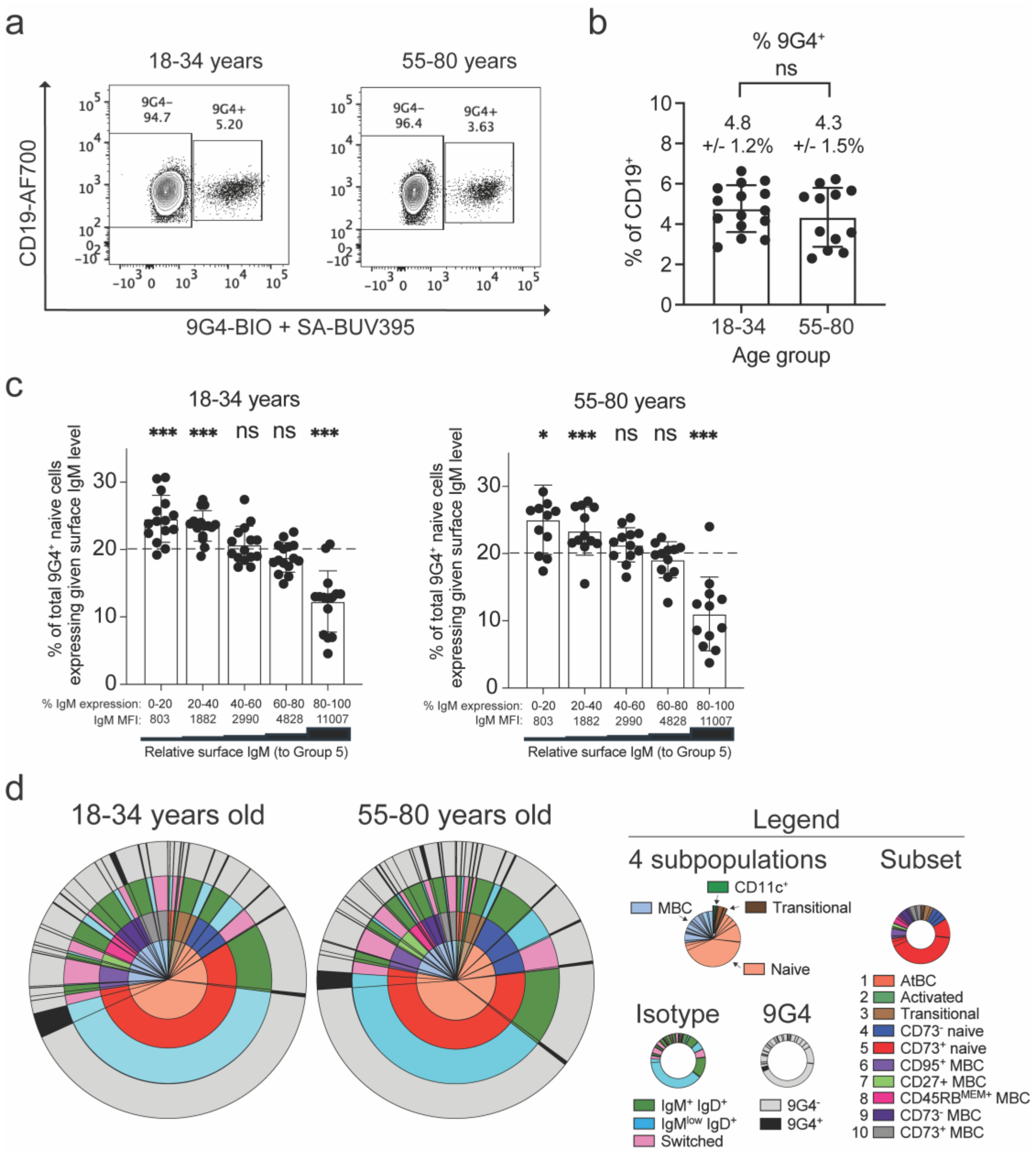

Supplementary Figure 6. Percentages of 9G4$^+$ B cells in younger (18-34 years) and older (55-80 years) individuals, related to Figure 4.

a. Representative flow cytometry plots of 9G4 staining on peripheral blood CD19$^+$ B cells are shown for the younger and older age groups.

b. A summary of 9G4$^+$ B cells is shown. Statistical significance was determined by p-values from non-parametric Mann-Whitney test, and p-value <0.05 was considered significant.

c. The percentage of 9G4$^+$ B_N cells within each group distinguished by surface IgM levels in 18-34 year (younger) and 55-80 year (older) individuals. Statistical significance was determined by Mann-Whitney test, and p-value <0.05 was considered significant, *p<0.05, **p<0.01, ***p<0.001.

d. A summary of the B cell populations from 18-34 and 55-80 year old individuals is shown in the target plots. The plots depict the four subpopulations (innermost), followed by the 10 subsets, isotypes, and 9G4 binding (outermost). B cells not expressing IgM or IgD are referred to as switched.

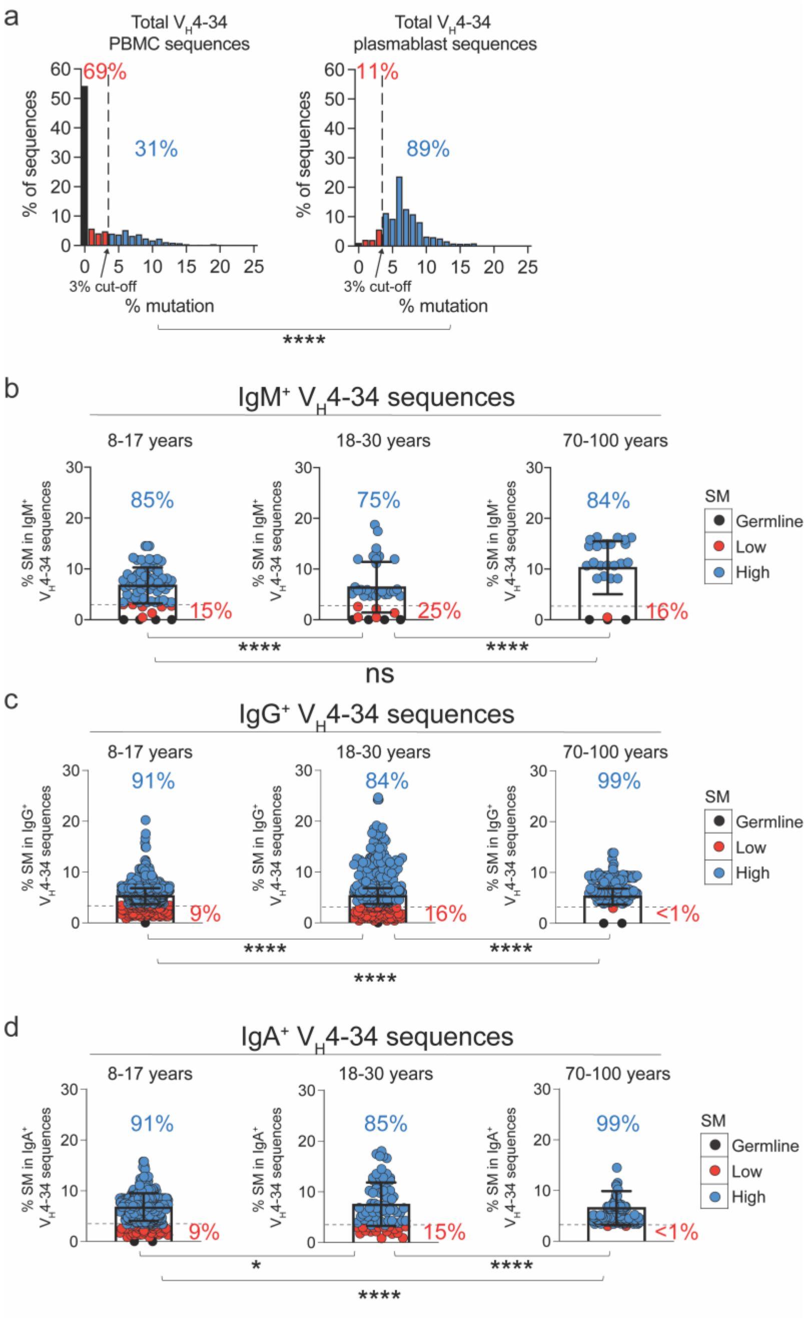

Supplementary Figure 7. Analysis of somatic mutations (SMs) at pre-immunization baseline and 7 days post-immunization with influenza vaccine, supplemental to Figure 5.
a. The percentage of V_H4-34 sequences in PBMCs at pre-immunization versus plasmablasts at 7 days post-immunization that were SM^{low} or SM^{high}. V_H4-34 sequences from all individuals in the study were combined for the analysis. The dotted line represents the cut-off of 3% SM, delineating SM^{Low} versus SM^{High}. The percentage of V_H4-34 sequences with SM^{High} were compared between PBMCs and plasmablasts were analyzed by Fisher's exact test. * p<0.05, ** p<0.01, *** p<0.001, **** p<0.0001, ns= not significant.
b-d. V_H4-34 IgM^+ (**b**), IgG^+ (**c**), and IgA^+ (**d**) sequences were grouped according to SM^{Low} versus SM^{High}. The dotted line represents the cut-off of 3% SM, delineating SM^{Low} versus SM^{High}. The percentage of SM^{High} sequences were compared between age groups and analyzed by Fisher's exact test. * p<0.05, ** p<0.01, *** p<0.001, **** p<0.0001, ns= not significant.

a

```
  1 caggtgcagc tacagcagtg gggcgcagga ctgttgaagc cttcggagac cctgtccctc
 61 agctgcgctg tctatggtgg gtccttcagt ggttactact ggaactggat ccgccagccc
121 ccagggaagg ggctggagtg gattggggaa atcaatcata gtggaagcac caactacaac
181 ccgtccctca agagtcgagt caccatatca gtagacacgt ccaagaacca gttgtccctg
241 aagctgagct ctgtgaccgc cgcggacacg gctgtgtatt actgtgcgag aggtgaaatt
301 gtagtagtac cagctgctag ctactactac tactacatgg acgtctggg
```

b

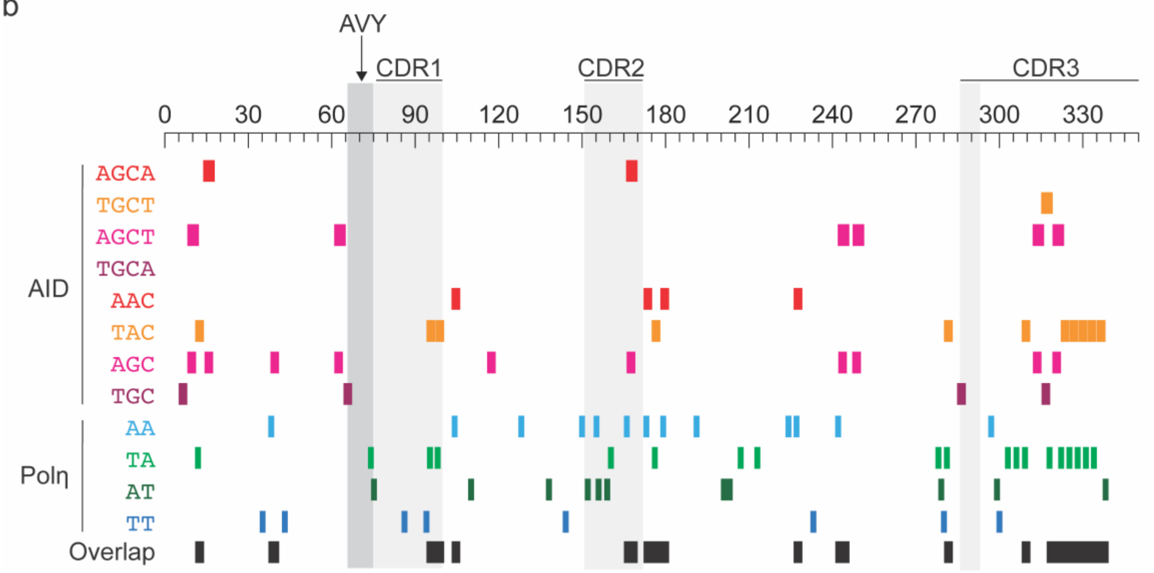

Supplementary Figure 8. Analysis of SM "hotspots" in the germline V_H4-34 DNA sequence.
a. The V_H4-34 DNA sequence from GenBank AJ564425.1 is shown.
b. The locations of the hotspots for activation-induced cytosine deaminase (AID) and DNA polymerase η (Polη) are summarized and color-coded. AID hotspots were identified by WGCW and WRC motifs, and Polη hotspots were identified by WA and TW motifs, where W=A/T and R=A/G. Overlapping AID and Polη hotspots are shown at the bottom in black. The AVY patch and CDR are indicated by the gray boxes.

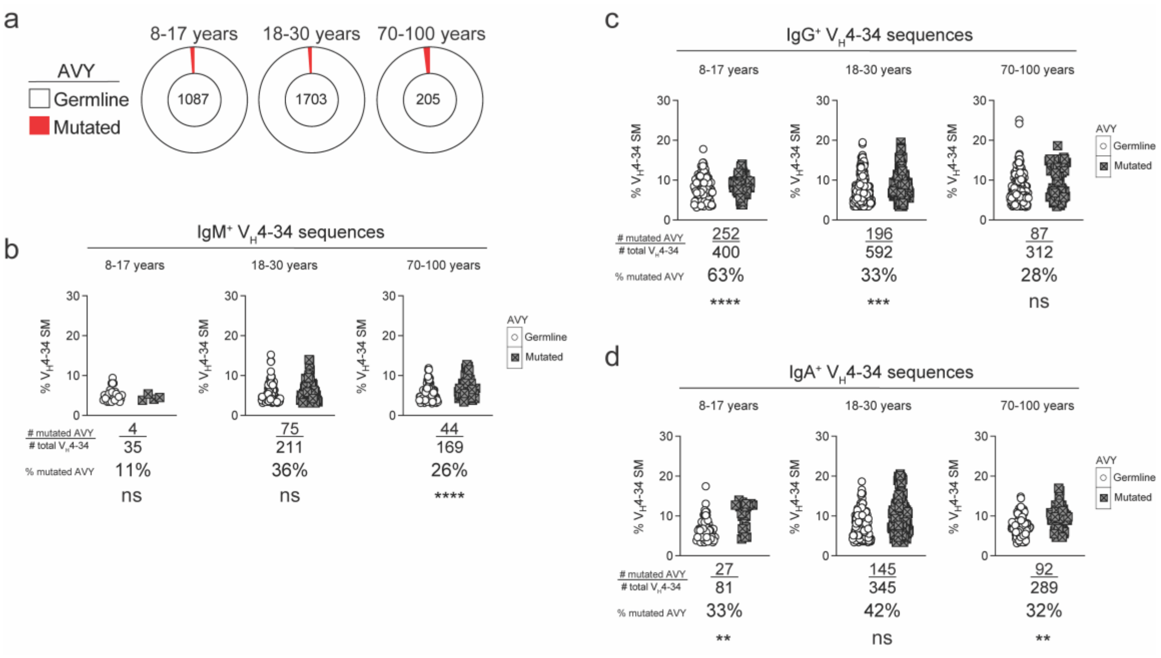

Supplementary Figure 9. Analysis of SM^{High} sequences and mutations at the AVY patch in V_H4-34 sequences from PBMCs at pre-immunization baseline, related to Figure 6.

a. Analysis of replacement mutations at the AVY patch of total V_H4-34 sequences from the 8-17, 18-30, and 70-100 year age groups at the pre-vaccination baseline timepoint. The number of V_H4-34 sequences analyzed in each group is shown in the middle of the doughnut plot.

b-d. V_H4-34 IgM$^+$ (**b**), IgG$^+$ (**c**), and IgA$^+$ (**d**) sequences from PBMCs that were SM^{high} (SM>3%) were analyzed for mutation at the AVY patch. For every age group and isotype, the percentage of V_H4-34 sequences with SM^{High} were compared to SM^{High} sequences from plasmablasts (Fig. 6a-c) analyzed by Fisher's exact test. ** $p<0.01$, *** $p<0.001$, **** $p<0.0001$, ns= not significant.

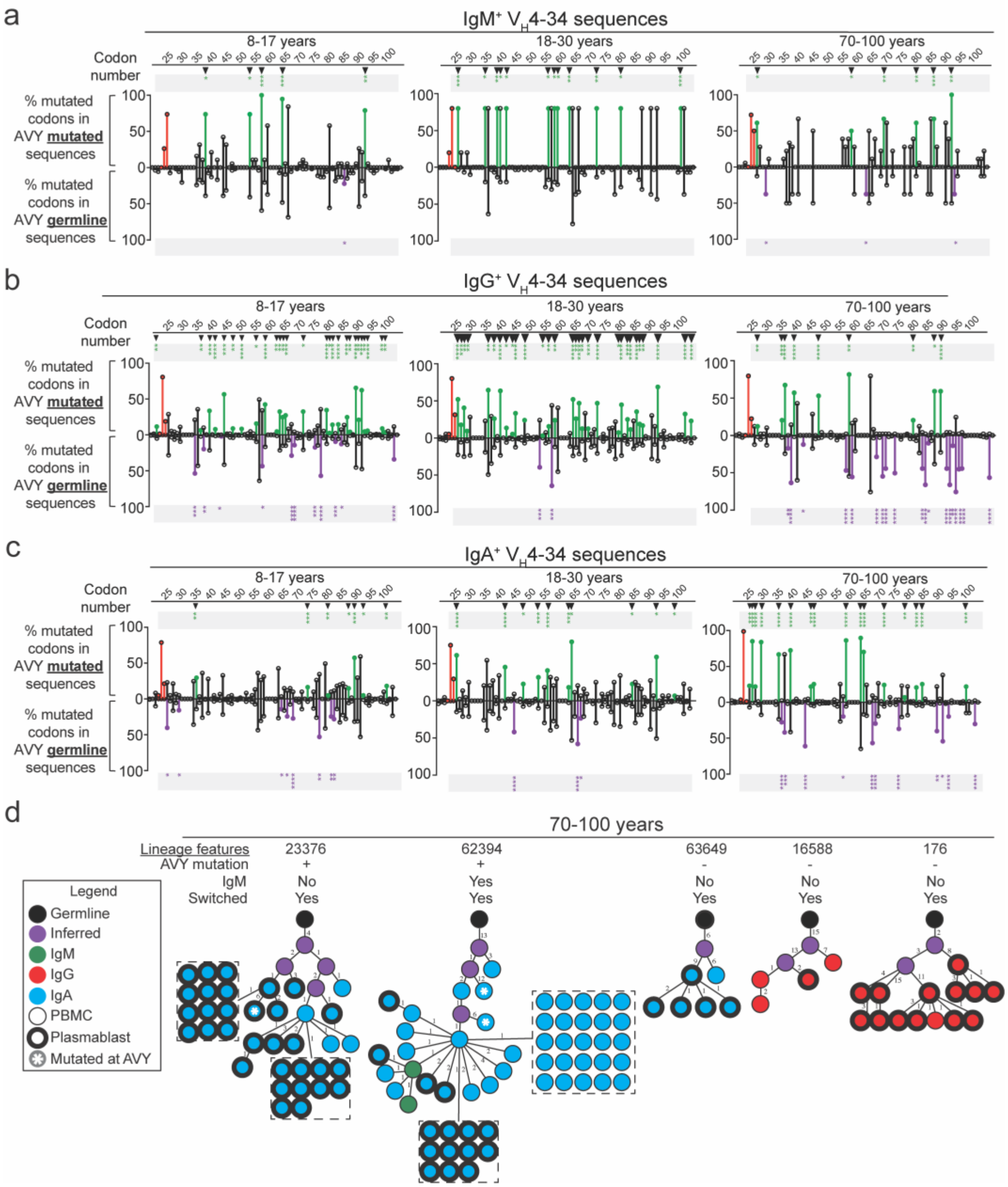

Supplementary Figure 10. Mutation and lineage analyses of V_H4-34 sequences from individuals immunized with influenza vaccine, related to Figure 6.

a-c. V_H4-34 IgM$^+$ (**a**), IgG$^+$ (**b**), and IgA$^+$ (**c**) plasmablast-derived sequences from 8-17, 18-30, and 70-100 year old vaccinees were grouped according to AVY germline (lower) versus AVY mutated (upper) (positions 23-25, in red), and the percentage of replacement mutations at each codon was analyzed for statistical significance by Fisher's exact test. Codons in which mutations were highly enriched in the AVY mutated sequences are emphasized by the arrowheads and are indicated in green. Codons in which mutations were highly enriched in the AVY germline sequences are indicated in purple. *p<0.05, **p<0.01, and ***p<0.001.

d. Given are the isotype distribution, cell phenotype, and mutation at the inherently autoreactive hydrophobic patch AVY for the V_H4-34 lineages from the 70-100 year age group. Each number corresponds to the clone identified from the analysis.

Donor	Biologic sex	Age (years)	Race	Age group
23	F	63	AA	Older
25	F	66	W	Older
27	M	69	W	Older
28	M	32	W	Younger
29	F	28	W	Younger
30	F	26	W	Younger
31	M	77	W	Older
34	F	27	W	Younger
35	M	60	W	Older
38	M	77	W	Older
41	M	68	A	Older
43	F	60	A	Older
44	F	28	W	Younger
45	F	19	L	Younger
47	M	25	W	Younger
50	M	73	W	Older
51	F	24	Mixed	Younger
53	M	71	W	Older
54	M	56	AA	Older
55	F	22	W	Younger
56	F	27	W	Younger
59	M	21	W	Younger
65	F	28	W	Younger
67	F	64	AA	Older
80	F	31	W	Younger
81	M	23	A	Younger
82	M	27	W	Younger

Supplementary Table 1. Demographics of subjects analyzed for surface marker phenotype by flow cytometry to determine B cell subpopulations, subsets, isotypes, and 9G4 binding, related to Figures 1, 2, 3, and 4. F= female, M= male, A=Asian, AA=African-American, L= Latin-American, W=White.

Age group	Biologic sex (n, %)	Age (years)	Race (n, %)
Younger	Female= 10/15, 67%<br>Male= 5/15, 33%	26 ± 4	A= 1, 7%<br>AA= 0, 0%<br>L= 1, 7%<br>Mixed= 1, 7%<br>White= 12, 80%
Older	Female= 4/12, 33%<br>Male= 8/12, 67%	66 ± 7	A= 2, 17%<br>AA= 3, 25%<br>L= 0, 0%<br>Mixed= 0, 0%<br>White= 7, 58%

Supplementary Table 2. Summary of subjects analyzed for surface marker phenotype by flow cytometry to determine B cell subpopulations, subsets, isotypes, and 9G4 binding, related to Figures 1, 2, 3, and 4. F= female, M= male, A=Asian, AA=African-American, L= Latin-American, W=White.

Donor	Biologic sex	Age (years)	Race
73	M	38	AA
79	F	48	AA
80	M	30	A
83	M	44	W

Supplementary Table 3. Demographics of subjects to measure B cell activation by calcium flux, related to Figure 3. F= female, M= male, A=Asian, AA=African-American, W=White.

Donor	Biologic sex	Age (years)	Race	Age group
66	F	64	W	Older
69	M	78	W	Older
74	F	32	W	Younger
75	F	31	W	Younger
76	M	25	W	Younger
77	M	64	W	Older
78	M	25	W	Younger
80	M	30	A	Younger
81	M	60	W	Older
82	F	65	W	Older

Supplementary Table 4. Demographics of subjects for analysis of PTEN expression in B_N cells, related to Figure 3. F= female, M= male, A=Asian, W=White.
